# In Vitro Ileal Fermentation is Affected More by the Fiber Source Fermented than the Ileal Microbial Composition in Growing Pigs

**DOI:** 10.1016/j.cdnut.2023.100076

**Published:** 2023-04-11

**Authors:** Anna ME. Hoogeveen, Paul J. Moughan, Suzanne M. Hodgkinson, Natascha Stroebinger, Wenjun Yu, Elizabeth A. Rettedal, Warren C. McNabb, Carlos A. Montoya

**Affiliations:** 1 Riddet Institute, Te Ohu Rangahau Kai, Massey University, Palmerston North, New Zealand; 2 School of Food and Advanced Technology, Massey University, Palmerston North, New Zealand; 3 Smart Foods and Bioproducts, AgResearch Limited, Te Ohu Rangahau Kai, Massey University, Palmerston North, New Zealand

**Keywords:** dietary fiber, ileal microbiota, in vitro ileal fermentation, pig, human

## Abstract

**Background:**

The fermentation of undigested material in the ileum is quantitatively important. However, the respective contributions of the microbial composition and the substrate to ileal fermentation are unclear.

**Objective:**

This aim was to investigate the contribution of microbial composition and fiber source to in vitro ileal fermentation outcomes.

**Methods:**

Thirteen ileal cannulated female pigs (Landrace/Large White; 9-wk-old; 30.5 kg body weight) were given diets containing black beans, wheat bread, chickpeas, peanuts, pigeon peas, sorghum, or wheat bran as the sole protein source for 7 d (100 g protein/kg dry matter diet). On day 7, ileal digesta were collected and stored at −80°C for microbial analysis and in vitro fermentation. For each diet, a pooled ileal inoculum was prepared to ferment different fiber sources (cellulose, pectin, arabinogalactan, inulin, fructooligosaccharides, and resistant starch) for 2 h at 37°C. Organic matter fermentability and organic acid production were determined following in vitro fermentation. Data were analyzed using a 2-way ANOVA (inoculum × fiber).

**Results:**

Forty-five percent of the identified genera in the digesta differed across diets. For instance, the number of *Lactococcus* was 115-fold greater (*P* ≤ 0.05) in the digesta of pigs fed the pigeon pea diet than for pigs fed the wheat bran diet. For both in vitro organic matter fermentability and organic acid production, there were significant (*P* ≤ 0.05) interactions between the inoculum and the fiber source. For instance, pectin and resistant starch resulted in 1.6- to 31-fold more (*P* ≤ 0.05) lactic acid production when fermented by the pigeon pea inoculum than other inocula. For specific fiber sources, statistically significant correlations were found between the number of bacteria from certain members of the ileal microbial community and fermentation outcomes.

**Conclusions:**

Both the fiber source fermented and the ileal microbial composition of the growing pig affected in vitro fermentation; however, the effect of the fiber source was predominant.

Curr Dev Nutr 2023;x:xx.

## Introduction

Studies using a combined in vivo/in vitro fermentation assay in the growing pig have shown that organic matter (OM) fermentation in the lower small intestine is quantitatively similar to that in the hindgut [1,2]. For example, the in vitro OM fermentability in pigs fed a human-type diet was 28% in the ileum and 35% in the hindgut, despite differences in microbial composition and fermentation time [1]. Organic acids, such as acetic and lactic acids, are found in the ileum of growing pigs [3] and adult humans [4]. These organic acids can be absorbed directly in the ileum [1,5] and have been found in the bloodstream of human ileostomates [6]. Ileal fermentation is quantitatively important and could have important implications for nutrition and health.

Foods (eg, cereals, pulses) commonly consumed by humans are diverse in both type and amount of fiber (such as β-glucan in cereals or pectin in fruits). It is known that the type and amount of dietary fiber can change the ileal microbial community composition in growing pigs [2,7,8] and adult humans [9,10] and influence in vitro ileal fermentation outcomes when using a porcine inoculum [2,11]. For example, the partial replacement of dietary cellulose with kiwifruit fiber resulted in a higher α-diversity of the ileal microbial community of growing pigs and a 38% decrease in acetic acid production [2]. However, it is unclear whether the ileal fermentation outcomes were due to changes in the microbial community composition (“inoculum”) or the nature of the undigested material entering the ileum (“substrate”).

It has been suggested that short-chain fatty acid (SCFA) production during in vitro human hindgut fermentation (based on a fecal inoculum) is primarily influenced by available substrate rather than interdifferences in the microbial community composition [12]. In contrast, it has been shown that *Prevotella*- and *Bacteroides*-dominant human fecal microbial communities have different in vitro fermentative capacities, resulting in different SCFA production for the fermentation of individual substrates [13]. Moreover, differences across substrates have been reported for in vitro fermentation using the same human fecal inoculum [13]. These studies show that hindgut fermentation is influenced by both the microbial community composition and the substrate, but the substrate appears to be the major contributor to human fecal fermentation [12,13]. The ileal microbial community composition of growing pigs [1,2] and adult humans [14,15] differs from that of the hindgut microbial community. The ileal microbiota is highly adapted to metabolize relatively simple sugars (mono-, di-, and oligo-saccharides) [15]. Still, it can also utilize complex polysaccharides [11,16,17].

Although it is now well established that there is an active ileal microbiota that ferments undigested material from the upper gastrointestinal tract (GIT), little is known about its fermentative capacity. This study aimed to investigate the effect of ileal microbial community composition and fiber source on in vitro ileal fermentation using the growing pig as a model for adult humans. Associations between fermentation outcomes and the number of specific ileal microbes were also determined. It was hypothesized that diets with different nutrient contents would drive changes in the ileal microbial community composition. These differences in the ileal microbial community composition and the fiber source fermented were hypothesized to affect in vitro ileal fermentation outcomes.

## Materials and Methods

### Diets

This study was part of a larger study to determine ileal amino acid digestibility in foods commonly consumed by humans [18]. The diets were balanced only for crude protein content, resulting in diets with a wide range of nutrient amounts (Supplementary Table S1). For example, the total dietary fiber content ranged from 57 to 295 g/kg DM diet. Previous studies have shown that dietary fiber modulates the ileal microbial community [2]. Thus, this study was a unique opportunity to collect terminal ileal digesta with different microbial communities to determine their contribution to in vitro ileal fermentation while applying the reduction principle of the 3Rs associated with animal experimentation.

For the current study, only 7 of 10 diets used in the larger study were selected. These 7 diets contained whole foods with varying nutrient content (black beans (*Phaseolus vulgaris*), bread (wheat, white), chickpeas (*Cicer arietinum*), peanuts (*Arachis hypogaea*; roasted), pigeon peas (*Cajanus cajan*), sorghum (*Sorghum bicolor*; flour), and wheat bran (Kellogg’s All-Bran)), whereas the other 3 diets contained a purified protein source and thus were similar in nutrient content. Each experimental diet was formulated to include one of the test foods as the sole protein source at a concentration of 100 g crude protein/kg DM diet and to meet the growing pigs’ nutrient requirements [19] except for crude protein and amino acids. The test foods were either bought ready to eat or prepared as detailed in the Supplementary Methods. Before feeding, each test food was combined with a mixture of non–protein-containing ingredients specific to each diet, containing maize starch, sucrose, vitamin–mineral premix, refined vegetable oil, and cellulose (Supplementary Table S1).

### In vivo study

#### Animals and experimental design

The ethical approval for this study was obtained from the Massey University Animal Ethics Committee (Protocol 16/121). The in vivo study was conducted according to the previously published protocol [20], with minor adaptations described below. Thirteen female pigs (Landrace/Large White; 9-wk-old; 30.5 ± 1.12 kg initial body weight (BW), mean ± SD) were individually housed in metabolic pens (1.5 × 1.5 m) in a room maintained at 21°C ± 2°C with a 12 h/12 h light/dark cycle. Each pig had a T-cannula surgically implanted at the terminal ileum (15 cm before the ileal cecal valve junction). After the surgery, the pigs had an 8- to 10-d recovery period, during which they received a basal diet. The pigs had free access to water for the whole duration of the study.

The parent study consisted of 9 experimental periods of 7 d each. The diets were randomly allocated during experimental periods 1–3 and 6–9 using an incomplete Latin Square design (*n* = 6 pigs/diet). The pigs received a protein-free and basal diet in periods 4 and 5, respectively. Pigs were given each a diet for 7 d with their daily ration (80 g DM/kg metabolic BW; BW^0.75^) divided into 2 equal-sized meals at 08:00 and 17:00. On day 7, ileal digesta were collected 3 h postfeeding (20:00). A plastic bag flushed with CO_2_ was attached to the ileal cannula using a rubber band until approximately 50 g of digesta was collected (between 15 min and 2 h). The collected digesta were mixed, and 2 aliquots were taken for microbial analysis. The aliquots and the remaining ileal digesta in the plastic bag were stored at −80°C. Supplementary Table S2 describes the ileal samples that were collected. Pigs were weighed weekly, and the daily ration was adjusted accordingly.

### In vitro ileal fermentation assay

For in vitro ileal fermentation, a methodology was used that has been optimized for several parameters such as pH, fermentation time, and inoculum concentration [21]. The amount of ileal digesta collected from individual pigs was insufficient to perform the in vitro fermentation assay for each pig, so a pooled inoculum was prepared for each diet by mixing the ileal digesta of all pigs fed the same diet (ie, an equal amount of wet weight digesta per pig). The inoculum was prepared by combining the pooled digesta (220 g) with 1 L of sterilized 0.1 M phosphate-buffered saline (PBS, anaerobic, 4.1 mM L-cystine, pH 7) at 37°C, homogenizing it for 15 s and straining the mixture through 4 sterilized layers of cheesecloth to remove large particles. PBS (5 mL) was added to the fermentation bottles containing no fiber (ie, blanks) or 100 mg fiber source, after which the bottles were flushed with CO_2_. Then, 5 mL of the ileal inoculum was added to the bottle. The bottle was flushed with CO_2_ again, capped, and incubated at 37°C for 2 h [21]. Ten fermentation bottles were used per fiber source or blank. The fiber sources used for the in vitro ileal fermentation were cellulose, pectin (from citrus peel), inulin (from chicory), (+)−arabinogalactan (AG; from larch wood), fructooligosaccharides (FOS; from chicory), and high-amylose corn starch (Hylon VII, Ingredion) containing 74% type 2 resistant starch [22]. All fiber sources, except resistant starch, were obtained from Sigma–Aldrich. Each inoculum had its own blanks to account for the potential fermentation of background OM in the inoculum (refer to calculations in the Supplementary Methods).

### Chemical analysis

The diets were analyzed for dry matter (DM), ash [23], crude protein (nitrogen × 6.25) using an elemental analyzer LECO, total lipids using a Soxhlet extractor and petroleum ether extraction, starch using a Total Starch Assay Kit (AA/AMG, Megazyme), total dietary fiber (including soluble and insoluble fiber) [24], and gross energy using a LECO AC-350 Automatic Calorimeter. After in vitro ileal fermentation, half the bottles (ie, *n* = 5) were analyzed for DM and ash [23] to determine OM fermentability. The OM fermentability in the samples was corrected by the OM present in the blanks (ie, no fiber source added, refer to calculations in the Supplementary Methods).

#### Organic acid analysis

The remaining bottles (*n* = 5) were used to determine the organic acid concentration of acetic, propionic, butyric, valeric, iso-butyric, iso-valeric, formic, lactic, and succinic acids using gas chromatography methodology [25]. Organic acids were derivatized using *N*-tert-butyldimethylsilyl-*N*-methyltrifluoroacetamide with 1% tert-butyldimethylchlorosilane (Sigma–Aldrich). 2-Ethylbutyric acid (5 mM) was used as an internal standard. The samples and standards were analyzed on a gas chromatograph (GC-2010, Shimadzu) equipped with an SH-Rtx-1 column (30 m × 0.25 mm × 0.25 μm; Shimadzu) and flame ionization detector, using helium as the carrier gas [26]. The organic acid production in the samples was corrected by the organic acids present in the blanks (ie, no fiber source added, refer to calculation in the Supplementary Methods). The concentrations of butyric, valeric, iso-butyric, and iso-valeric acids were negligible in the samples (ie, below the detection limit). Thus, the total organic acid production was calculated as the sum of formic, acetic, propionic, lactic, and succinic acid concentrations.

### Microbial analysis

A Qiagen PowerSoil kit (Qiagen) was used to extract the DNA from ileal digesta for each pig. The manufacturer’s instructions were followed with minor adjustments described previously [2]. The total number of bacteria (ie, 16S rRNA gene copies) was determined using a qPCR, and Illumina sequencing was performed to determine the taxonomic composition (Supplementary Methods).

### Statistical analysis

A sample size of 6 animals was available from the parent study. This sample size was also sufficient to reach a statistically significant difference in the ileal microbial composition between diet groups with a power >80% at a 2-tail 5% significance level based on the previously reported means and variances [2]. For in vitro ileal fermentation, between 2 and 6 replicates (fermentation bottles) were required to detect a statistical difference with a power >80% at a 2-tail 5% significance level based on reported means and variance [21]. Five technical replicates using ileal digesta pooled across animals within the same diet were used for each diet and fiber source combination. For the microbial analysis, the number of replicates (ie, animals) differed per diet because, for some of the samples, the extracted DNA had a concentration or quality that was too low to obtain accurate 16S rRNA gene sequencing results.

The significant separation for the groupings of the ileal samples after performing the Bray–Curtis similarity, principal coordinates analysis (PCoA) was tested in Calypso using the Adonis permutational (999 permutations) and betadisper tests. The remaining statistical analyses were performed using the SAS software (version 9.4, SAS Institute Inc.). Only microbial taxa with more than 1% relative abundance in at least 1 sample were included in the statistical analyses. Thus, for the taxonomic composition (number of gene copies per gram ileal digesta), Shannon diversity, and predicted metabolic activity, the effect of the diet was analyzed with a 1-way ANOVA model using the Proc Mixed procedure. For the taxonomic composition, Shannon Diversity Index, and predicted metabolic activity data, the effect of diet was included in the model as a fixed effect and the period and diet sequence as random effects, with the pig as the experimental unit. For all the response variables, period and diet sequence did not have (*P* > 0.05) a significant effect and therefore were removed from the model.

Some bacterial taxa were not detected or detected in only a few pigs fed the same diet. Therefore, next to the taxonomic composition analysis, a frequency analysis was also performed using binary logistic regression using the Proc Glimmix procedure with 0 when the taxon was not present and 1 when a taxon had at least 1 read. No statistical analysis was performed if the frequency was 1 for all diets.

A 2-way ANOVA model was used to determine the effect of the inoculum, fiber source, and their interaction on the OM fermentability and production of total organic acids, formic, acetic, propionic, and lactic acids using the Proc Mixed procedure. Succinic acid production was observed for only 1 fiber source (ie, AG). Thus, a 1-way ANOVA model was used to test the effect of the inoculum on succinic acid production. The *F* value and the range mean values were used to determine the contribution of the substrate and inoculum to the ileal fermentation outcomes.

A combination of the ODS Graphics, Proc Univariate procedure, and the repeated statement of SAS was used to test the model diagnostics of each response variable. The taxonomic composition data and predicted metabolic activity were log_10_ transformed to achieve homogenous variance. The repeated statement in the Proc Mixed model allowed testing for the homogeneity of variance by fitting models with the Restricted Maximum Likelihood method and comparing them using the log-likelihood ratio test. The selected model for all response variables had similar Studentized residuals (ie, equal variances) across treatments. When the *F* value of the model was significant (*P* ≤ 0.05), the mean values were compared using the adjusted Tukey–Kramer test. Probability values were considered statistically different when *P* ≤ 0.05, and values of 0.05 < *P* < 0.10 were considered a trend.

Pearson correlation analyses were performed using the Proc Corr procedure to evaluate the relationship between fermentation outcomes (ie, OM fermentability and organic acid production) and the number of bacteria in different bacterial taxa in the inoculum (*n* = 7 inocula per fiber source). It was assumed that the number of bacteria in different bacterial taxa in the inoculum was the same as the average number of bacteria of the different bacterial taxa found in the ileal digesta used to prepare the inoculum (ie, ileal digesta from individual pigs receiving the same diet).

## Results

All pigs remained healthy, with daily live weight gain of 501 ± 25 g/d (mean ± SEM). One pig was excluded from the analysis as it displayed coprophagic behavior.

### In vitro OM fermentability

On average, the in vitro ileal OM fermentation of the blanks for all inocula was 0%, except for the peanut inoculum (4.2% ± 0.44%).

The in vitro ileal OM fermentability of the different added fiber sources ranged from 0% to 37% across all inocula. In general, pectin was significantly more (27.1% ± 0.39% on average across all inocula; *P* < 0.001; Table 1) fermented than the other fiber sources, whereas cellulose was significantly less fermented (0.35% ± 0.20%). There was a marked effect of the inoculum (microbial composition). The peanut inoculum supported a significantly greater in vitro ileal OM fermentability (19.1% ± 0.52% on average across all fiber sources; *P* ≤ 0.05) than the other inocula, and the sorghum inoculum had significantly lower OM fermentability (10.7% ± 0.39%). When considering all data together, an interaction (*P* < 0.001) between the inoculum and fiber source was observed for ileal OM fermentability. For example, FOS was, on average, 2.6-fold more (*P* ≤ 0.05) fermented by the black bean, bread, and peanut inocula than the chickpea, pigeon pea, sorghum, and wheat bran inocula. On the other hand, the black bean inoculum had a 2.1-fold higher (*P* ≤ 0.05) OM fermentability of resistant starch than the pigeon pea inoculum.

**TABLE 1:** In vitro organic matter fermentability of dietary fiber substrates fermented using pooled ileal inocula from growing pigs fed diets containing different test foods^1^

Inoculum	Fiber substrate						Mean (range)
	AG	Cellulose	FOS	Inulin	Pectin	High-amylose starch	
*%*							
Black bean	16.5 ± 1.11^bc,†‡^	0.0 ± 0.21^d,§^	21.8 ± 0.65^b,†^	19.4 ± 1.35^bc,†‡^	26.5 ± 0.52^a,‡^	14.8 ± 1.32^c,†^	16.4 (0.0–28.3)
Bread	17.5 ± 0.77^b,†‡^	1.1 ± 0.41^d,‡§^	28.7 ± 3.09^ab,†^	24.1 ± 1.45^ab,†^	25.2 ± 1.21^a,†‡^	9.3 ± 0.91^c,†‡^	17.7 (0.5–37.1)
Chickpea	13.9 ± 1.29^bc,†‡^	3.1 ± 0.58^d,†‡^	12.8 ± 0.50^b,‡^	12.1 ± 1.04^bc,‡§^	25.3 ± 1.71^a,†‡^	8.9 ± 0.69^c,†‡^	12.7 (2.0–29.1)
Peanut	22.5 ± 1.80^ab,†^	6.1 ± 0.38^d,†^	28.8 ± 2.25^a,†^	16.4 ± 0.75^b,‡^	30.1 ± 0.54^a,†^	10.5 ± 0.76^c,†‡^	19.1 (5.2–35.6)
Pigeon pea	15.9 ± 0.74^b,†‡^	0.0 ± 1.03^d,§^	8.9 ± 0.41^c,§^	12.8 ± 1.20^bc,‡§^	26.9 ± 0.61^a,†‡^	6.9 ± 0.51^c,‡^	11.0 (0.0–28.3)
Sorghum	15.3 ± 1.26^b,†‡^	0.0 ± 0.33^d,§^	7.4 ± 0.50^c,§^	6.2 ± 1.00^c,§^	29.1 ± 1.05^a,†‡^	6.9 ± 1.23^c,†‡^	10.7 (0.0–31.0)
Wheat bran	13.5 ± 0.79^b,‡^	0.0 ± 0.32^c,§^	12.1 ± 1.26^b,‡§^	8.1 ± 0.90^b,§^	26.6 ± 1.00^a,†‡^	10.1 ± 0.63^b,†‡^	11.6 (0.0–29.1)
Mean (range)	16.4 (10.1–29.1)	0.35 (0.00–7.03)	17.2 (6.3–37.1)	14.2 (4.4–28.8)	27.1 (19.4 –31.3)	9.6 (3.9–18.3)	
	Inoculum (I)	Substrate (S)	I × S				
*F* value	58.0	930	19.3				
*P* value	<0.001	<0.001	<0.001				

1 Values are mean ± SEM per inoculum, *n* = 5 fermentation bottles prepared after pooling ileal digesta of 6 ileal cannulated pigs. When negative values were observed (due to correction by OM found in the blanks), values were assumed to be zero, meaning that OM was not fermented). A 2-way ANOVA model was used to assess the effect of inoculum, fiber substrate, and their interaction. A repeated statement for each fiber substrate by inoculum combination was required to have similar Studentized residuals as described in the statistical analysis section. Data in a row (ie, fiber substrate effect) with different letters differ (*P* ≤ 0.05), and means in a column (ie, inoculum effect) with different symbols differ (*P* ≤ 0.05). AG, arabinogalactan; FOS, fructooligosaccharides; OM, organic matter.

### In vitro ileal organic acid production

Across all inocula, AG supported a significantly greater (*P* ≤ 0.05) total organic acid production after in vitro ileal fermentation than other fiber sources. At the same time, cellulose had a significantly lower (*P* ≤ 0.05) total organic acid production (Table 2). Across all fiber sources, a significantly greater (*P* ≤ 0.05) total organic acid production was obtained with the pigeon pea inoculum compared with the other inocula. When considering all data together for a total, formic, acetic, propionic, and lactic acid production, consistent with the OM fermentability results, there was an interaction between the inoculum and fiber source (*P* < 0.001). For example, when fermenting FOS, the peanut inoculum produced 2.5-fold higher total organic acids than the wheat bran inoculum. In comparison, the wheat bran inoculum produced 4.3-fold greater total organic acids when fermenting inulin than the peanut inoculum. Depending on the fiber source, some inocula did not produce propionic acid (Supplementary Table S3). For example, the peanut inoculum produced propionic acid when fermenting FOS but not when fermenting inulin. Succinic acid was only produced when AG was fermented.

**TABLE 2:** Production of total organic, formic, acetic, and lactic acids during in vitro fermentation of dietary fiber substrates using pooled ileal inocula from growing pigs fed diets containing different test foods^1^

Organic acid	Inoculum	Fiber substrate						
		AG	Cellulose	FOS	Inulin	Pectin	High-amylose starch	Mean (range)
mmol/kg DM substrate								
Total	Black bean	384 ± 10.6^a,‡^	31 ± 0.9^d,§^	69 ± 11.9^bcd,†‡§^	66 ± 5.0^c,‡^	114 ± 5.3^b,‡^	127 ± 6.6^b,†‡§^	132 (30–402)
	Bread	456 ± 15.8^a,†‡^	50 ± 1.0^c,‡^	99 ± 5.5^b,†‡^	90 ± 4.3^b,†‡^	37 ± 2.2^d,‖^	94 ± 11.6^bcd,‡§^	138 (33–506)
	Chickpea	502 ± 8.20^a,†^	55 ± 3.9^c,†‡^	110 ± 4.7^b,†^	89 ± 3.9^b,†‡^	42 ± 13.1^cbc,§‖^	97 ± 3.1^b,§^	149 (19–518)
	Peanut	480 ± 27.1^a,†‡^	77 ± 2.6^c,†^	130 ± 6.1^b,†^	33 ± 7.0^d,§^	96 ± 15.9^bc,‡§‖^	124 ± 3.9^b,‡^	157 (19–547)
	Pigeon pea	477 ± 24.0^a,†‡^	75 ± 7.4^c,†‡^	105 ± 10.1^c,†‡§^	62 ± 8.7^c,†‡§^	197 ± 7.0^b,†^	174 ± 8.6^b,†^	182 (48–535)
	Sorghum	402 ± 11.2^a,‡^	120 ± 13.4^b,†^	48 ± 10.9^b,‡c^	116 ± 15.9^b,†‡§^	78 ± 5.6^b,‡§^	102 ± 13.1^b,†‡§^	144 (27–436)
	Wheat bran	525 ± 33.1^a,†‡^	38 ± 8.6^cd,‡§^	52 ± 4.5^c,§^	141 ± 13.9^b,†^	18 ± 4.4^d,‖^	135 ± 10.9^b,†‡§^	152 (10–581)
	Mean (range)	461 (356–581)	64 (16–133)	88 (27–141)	85 (19–179)	83 (10–216)	122 (73–200)	
Formic acid	Black bean	323 ± 17.8^a^	3.5 ± 2.87^c,§^	8.7 ± 2.43^c,‡§^	5.6 ± 4.48^c,§^	8.2 ± 1.51^c,‡^	28.3 ± 3.84^b,‡§^	62.8 (3.00–337)
	Bread	324 ± 15.9^a^	29.2 ± 2.87^b,†^	16.3 ± 2.43^b,†‡§^	21.0 ± 3.47^b,‡§^	2.3 ± 2.14^c,‡^	27.8 ± 3.84^b,‡§^	70.1 (2.07–351)
	Chickpea	304 ± 15.9^a^	6.6 ± 2.03^c,§^	26.8 ± 2.11^b,†^	6.7 ± 5.49^bc,§^	3.4 ± 2.14^c,‡^	28.8 ± 2.72^b,‡^	62.7 (3.16–319)
	Peanut	348 ± 15.9^a^	7.2 ± 2.03^c,§^	26.8 ± 2.11^b,†^	17.0 ± 3.47^bc,‡§^	9.6 ± 1.65^c,‡^	31.2 ± 2.43^b,‡^	73.2 (3.40–385)
	Pigeon pea	323 ± 15.9^a^	21.4 ± 2.03^b,†‡^	7.3 ± 2.11^c,‡§^	7.3 ± 4.48^bc,§^	10.8 ± 1.51^bc,‡^	9.2 ± 3.14^bc,§^	63.1 (4.48–343)
	Sorghum	348 ± 15.9^a^	3.7 ± 4.06^e,‡§^	26.1 ± 2.98^d,†‡^	56.4 ± 3.88^bc,†^	41.3 ± 1.74^c,†^	76.3 ± 2.72^b,†^	92.0 (3.68–398)
	Wheat bran	375 ± 15.9^a^	10.9 ± 2.03^c,‡§^	11.0 ± 2.43^c,‡§^	37.6 ± 3.47^b,†‡^	11.4 ± 1.74^c,‡^	43.1 ± 2.43^b,‡^	81.6 (7.01–447)
	Mean (range)	335 (284–447)	11.8 (3.00–34.7)	17.6 (4.48–33.3)	21.7 (5.02–80.7)	12.4 (2.07–46.3)	35.0 (7.11–80.1)	
Acetic acid	Black bean	58.1 ± 1.48^a,§^	4.2 ± 2.10^c,§^	23.8 ± 1.71^b,‡§^	8.5 ± 1.71^c,§^	11.6 ± 1.71^c,‡§^	14.6 ± 1.48^bc,‡^	20.1 (3.89–64.4)
	Bread	44.0 ± 1.96^a,‖^	7.3 ± 3.11^b,§^	40.8 ± 3.11^a,†^	19.2 ± 2.54^b,‡§^	21.0 ± 1.96^b,‡^	20.4 ± 2.54^b,†‡^	25.4 (7.09–47.7)
	Chickpea	70.8 ± 2.09^a,†‡^	12.8 ± 2.70^d,‡§^	52.3 ± 2.34^b,†^	43.0 ± 2.70^bc,†^	2.01 ± 3.31^d,§^	33.3 ± 2.34^c,†^	35.7 (1.98–77.9)
	Peanut	72.6 ± 4.57^a,†‡§^	26.5 ± 5.89^b,‡§^	14.7 ± 5.10^b,‡^	14.4 ± 5.89^b,‡§^	43.3 ± 4.57^b,†^	23.0 ± 5.10^b,†‡^	32.4 (10.8–85.1)
	Pigeon pea	88.9 ± 3.76^a,†^	30.8 ± 3.76^b,‡^	39.9 ± 4.20^b,†‡§^	2.8 ± 4.20^c,§^	30.0 ± 4.20^b,†‡^	17.4 ± 4.20^bc,†‡^	35.0 (1.55–97.6)
	Sorghum	17.1 ± 10.14^b,‖^	123.0 ± 10.1^a,†^	25.6 ± 10.1^b,†‡§^	47.3 ± 7.85^b,†‡§^	19.9 ± 12.4^b,†‡§^	37.8 ± 14.2^b,†‡^	45.2 (11.2–152)
	Wheat bran	60.5 ± 3.56^a,‡§‖^	20.2 ± 4.11^b,‡§^	7.4 ± 5.03^b,§^	19.9 ± 4.11^b,‡§^	12.1 ± 7.12^b,†‡§^	29.3 ± 3.18^b,†‡^	24.9 (4.09–74.9)
	Mean (range)	58.9 ± 1.81 (14.7–97.6)	32.1 ± 1.97 (3.9–152)	29.2 ± 1.97 (4.1–58.8)	22.1 ± 1.74 (1.6–70.0)	20.0 ± 2.31 (2.0–61.8)	25.1 ± 2.13 (10.9–45.4)	
Lactic acid	Black bean	11.0 ± 6.66^c,‖^	22.6 ± 6.66^bc,†‡§^	32.4 ± 4.71^bc,†‡^	49.1 ± 4.71^b,†‡^	91.6 ± 6.66^a,‡^	95.2 ± 4.21^a,‡^	50.3 (9.1–113)
	Bread	80.4 ± 4.50^a,‡^	6.2 ± 5.03^d,§^	40.2 ± 4.50^bc,†^	47.4 ± 4.50^b,†‡^	14.0 ± 4.50^cd,‖#^	36.6 ± 4.50^bc,‖^	37.5 (4.2–96.5)
	Chickpea	119.0 ± 3.51^a,†^	21.8 ± 4.96^c,†‡§^	26.5 ± 3.51^c,†‡^	49.9 ± 4.05^d,†^	63.3 ± 7.02^b,‡§^	31.8 ± 3.14^bc,‖^	52.0 (19.2–122)
	Peanut	52.2 ± 4.68^a,§^	42.0 ± 4.68^ab,†^	7.1 ± 6.05^c,‡§^	15.8 ± 7.41^bc,‡§^	45.1 ± 6.05^ab,§‖^	11.3 ± 5.24^c,‖#^	28.9 (3.6–68.9)
	Pigeon pea	80.1 ± 9.33^b,†‡§^	43.2 ± 10.80^b,†‡§^	42.6 ± 10.7^b,†‡§^	54.7 ± 9.33^b,†‡^	155.0 ± 9.33^a,†^	152.0 ± 8.34^a,†^	88.1 (38.4–181)
	Sorghum	53.8 ± 1.63^a,§^	22.8 ± 1.89^b,†‡^	4.4 ± 1.63^d,§^	6.1 ± 1.63^d,§^	17.5 ± 1.63^bc,#^	9.11 ± 1.89^cd,#^	18.9 (2.1–58.3)
	Wheat bran	58.7 ± 2.93^a,‡§^	15.1 ± 3.68^c,‡§^	38.2 ± 2.93^b,†^	38.3 ± 3.38^b,†‡^	5.0 ± 2.93^c,#^	65.6 ± 2.93^a,§^	368 (1.5–73.2)
	Mean (range)	65.0 (9.1–122)	24.8 (4.2–48.2)	27.4 (2.1–50.8)	37.3 (5.1–76.1)	56.0 (1.5–181)	57.4 (7.6–175)	
*F* value		Inoculum (I)	Substrate (S)	I x S				
	Total	13.6	489	31.3				
	Formic acid	13.3	567	10.0				
	Acetic acid	35.2	58.3	16.6				
	Lactic acid	120	66.8	35.4				
*P* value		Inoculum (I)	Substrate (S)	I × S				
	Total	<0.001	<0.001	<0.001				
	Formic acid	<0.001	<0.001	<0.001				
	Acetic acid	<0.001	<0.001	<0.001				
	Lactic acid	<0.001	<0.001	<0.001				

1 Values are mean ± SEM, *n* = 5 fermentation bottles prepared after pooling ileal digesta of 6 ileal cannulated pigs. A 2-way ANOVA model was used to assess the effect of inoculum, fiber substrate, and their interaction for total organic, formic, acetic, and lactic acids. A different repeated statement was required for each organic acid to have similar Studentized residuals, as described in the statistical analysis section. For example, the best-repeated statement for formic acid had a common SEM per fiber substrate, whereas for acetic acid, it had a common SEM per inoculum. Means in a row (ie, inoculum effect) with different letters differ (*P* ≤ 0.05), and means in a column (ie, fiber substrate effect) with different symbols differ (*P* ≤ 0.05). The butyric, valeric, iso-butyric, and iso-valeric acid production were negligible in the samples (ie, below the detection limit) and, therefore, not reported. The means of propionic and succinic acid production are reported in Supplementary Table S3. AG, arabinogalactan; DM, dry matter; FOS, fructooligosaccharides; ND, not detected.

### Ileal microbial composition

In the current study, 7 phyla and 57 genera were identified (>1% relative abundance in at least 1 sample). Six phyla (86%) and 27 genera (47%) were present in ileal digesta for all diets at a 100% frequency. The remaining taxa displayed different frequencies across the diets (Supplementary Table S4). For example, *Leuconostoc* had a higher (0.83 vs. < 0.25; *P* ≤ 0.05) frequency in the ileal digesta of pigs fed the sorghum diet than in those given the bread, chickpea, peanut, or wheat bran diets.

The pigs fed the bread diet had a 3.5-fold higher total number of bacteria in their ileal digesta than pigs fed the sorghum and wheat bran diets (based on back-transformed data; Table 3). Forty-five percent of the total genera identified differed (*P* ≤ 0.05) across diets (Supplementary Table S5). For example, the number of Proteobacteria was on average 3.3-fold higher (*P* ≤ 0.05) in the ileal digesta of pigs fed the bread and sorghum diets than those given the wheat bran diet (based on back-transformed data). Based on PICRUSt, the predicted metabolic activity of the ileal microbial composition of pigs was similar across all diets, with a few exceptions (Supplementary Table S6).

**TABLE 3:** Effect of diet on the number of bacteria in ileal digesta from growing pigs fed diets containing different test foods^1^

Phylum	Genera	Diet							SEM	*P* value
		Black bean	Bread	Chickpea	Peanut	Pigeon pea	Sorghum	Wheat bran		
Sample size, *n*^2^		4	5	4	5	6	6	6		
log_10_ 16S rRNA gene copies/g wet digesta										
Total bacteria		10.6^ab^	11.1^a^	10.8^ab^	10.9^ab^	11.1^ab^	10.6^b^	10.3^b^	0.180	0.002
Actinobacteria										
*Actinomyces*		7.67^ab^	8.32^ab^	7.95^ab^	7.82^ab^	8.30^a^	7.66^ab^	7.43^b^	0.192	0.036
*Bifidobacterium*		7.73^a^	6.77^ab^	8.29^ab^	7.34^ab^	7.71^ab^	5.98^b^	6.87^ab^	0.556	0.009
*Collinsella*		6.99^ab^	7.61^a^	6.68^ab^	7.74^ab^	7.11^ab^	6.04^b^	7.47^a^	0.357	0.010
Bacteroidetes		9.79^b^	10.3^a^	9.87^ab^	10.3^ab^	10.2^ab^	9.59^ab^	9.83^ab^	0.164	0.020
*Bacteroides*		9.29^ab^	9.79^a^	9.33^ab^	9.81^ab^	9.67^ab^	9.17^ab^	8.64^b^	0.229	0.038
*Parabacteroides*		7.47^b^	8.23^a^	7.50^abc^	7.79^abc^	7.92^abc^	7.89^ab^	6.73^c^	0.252	0.001
*Prevotella*		9.29^ab^	9.81^a^	9.29^ab^	9.45^ab^	9.89^ab^	8.93^b^	9.71^a^	0.205	0.009
Firmicutes		10.3^ab^	10.9^a^	10.6^ab^	10.5^ab^	10.8^ab^	10.2^b^	9.85^b^	0.200	0.005
*Agathobacter*		7.15^ab^	7.52^ab^	6.93^ab^	7.28^ab^	6.54^ab^	6.14^b^	7.86^a^	0.352	0.027
*Anaerovibrio*		6.66^bc^	7.40^b^	7.39^ab^	8.09^abc^	7.10^abc^	5.74^c^	8.82^a^	0.398	<0.001
*Blautia*		7.25^b^	7.44^ab^	7.17^ab^	7.81^ab^	7.45^ab^	7.18^b^	8.47^a^	0.329	0.008
*Clostridium_sensu_stricto_1*		8.54^ab^	10.1^a^	9.53^ab^	9.62^ab^	9.53^ab^	8.98^b^	8.13^c^	0.309	0.035
*Enterococcus*		8.63^a^	8.13^a^	6.85^b^	7.65^ab^	8.47^ab^	7.62^a^	7.96^ab^	0.379	0.007
*Faecalibacterium*		6.54^bc^	6.47^bc^	6.57^bc^	6.57^bc^	6.61^b^	5.69^c^	8.44^a^	0.315	<0.001
*Fusicatenibacter*		6.08^b^	6.14^b^	5.84^b^	ND	6.12^b^	5.81^b^	7.71^a^	0.241	0.027
*Lachnospiraceae_unclassified*		7.98^b^	8.63^a^	8.22^ab^	8.71^ab^	8.56^ab^	8.30^ab^	8.32^ab^	0.192	0.019
*Lactococcus*		7.37^ab^	6.66^ab^	6.84^ab^	6.19^ab^	7.85^a^	7.97^a^	5.79^b^	0.432	0.007
*Megamonas*		7.62^ab^	7.77^abc^	7.95^ab^	6.79^bc^	7.63^abc^	5.96^c^	8.79^a^	0.371	<0.001
*Megasphaera*		7.02^ab^	7.14^a^	8.19^ab^	7.32^ab^	7.67^a^	5.67^b^	6.82^a^	0.486	0.001
*Romboutsia*		8.61^abc^	9.81^a^	9.26^ab^	9.24^ab^	9.41^ab^	9.07^b^	7.24^c^	0.236	0.001
*Sarcina*		9.09^ab^	10.3^a^	8.37^ab^	8.18^ab^	9.91^a^	8.30^ab^	6.54^b^	0.554	0.003
*Selenomonadaceae_unclassified*		7.33^abc^	9.35^abc^	9.66^a^	7.37^c^	9.43^ab^	7.42^abc^	7.70^bc^	0.376	0.019
*Streptococcus*		9.45^ab^	9.80^a^	9.31^ab^	9.22^ab^	9.49^ab^	9.23^ab^	8.75^b^	0.290	0.039
*Terrisporobacter*		8.60^ab^	9.62^a^	9.26^a^	8.91^a^	8.63^ab^	8.87^a^	7.16^b^	0.330	<0.001
*Turicibacter*		9.13^ab^	9.52^a^	9.54^a^	9.67^a^	9.45^a^	8.68^a^	7.58^b^	0.324	<0.001
*Veillonella*		8.65^ab^	9.19^ab^	9.28^a^	8.77^ab^	9.36^ab^	8.11^b^	8.78^ab^	0.297	0.030
Fusobacteria		9.43^b^	10.0^a^	9.37^b^	10.0^ab^	9.79^ab^	9.36^b^	9.16^b^	0.166	0.002
*Fusobacterium*		9.42^b^	10.0^a^	9.36^b^	10.0^ab^	9.76^ab^	9.35^b^	9.16^b^	0.164	0.002
Proteobacteria		10.0^ab^	10.1^a^	9.59^ab^	10.1^ab^	10.2^ab^	10.0^a^	9.56^b^	0.180	<0.001
*Acinetobacter*		7.28^ab^	7.32^ab^	6.24^b^	6.77^ab^	6.87^ab^	8.31^a^	6.73^ab^	0.293	0.009
*Enterobacterales_unclassified*		7.58^ab^	7.82^a^	7.32^ab^	7.80^ab^	7.84^ab^	7.91^ab^	7.12^b^	0.223	0.013
*Kosakonia*		6.99^ab^	6.46^ab^	ND	6.06^ab^	7.36^ab^	7.43^a^	5.36^b^	0.458	0.009
*Pseudomonas*		5.77^bc^	6.26^b^	ND	6.22^bc^	6.49^ab^	8.50^a^	5.37^c^	0.269	0.001
*Sutterella*		7.86^ab^	8.43^ab^	8.11^ab^	8.67^a^	8.31^ab^	7.19^b^	8.17^ab^	0.326	0.035

1 Values are mean with pooled SEM, *n* = 4-6 animals per diet. Only taxa with >1% relative abundance in at least 1 of the samples and that showed a significant diet treatment effect (Supplementary Table S5) are presented. The number of 16S rRNA gene copies per taxa was obtained by multiplying the total number of 16S rRNA gene copies with the relative abundance of the taxa with the assumption that each taxon has an equal number of 16S rRNA gene copies. Data were log_10_ transformed to achieve homogenous variance. A 1-way ANOVA model was used to assess the effect of the diet. Means in a row with a different letter differ (*P* < 0.05). ND, not detected.

2 *n* Indicates the number of replicates. The different numbers of replicates resulted from either removing 1 pig that displayed coprophagy or the extracted DNA having low quality or concentration for 16S rRNA gene sequencing.

**FIGURE 1:**
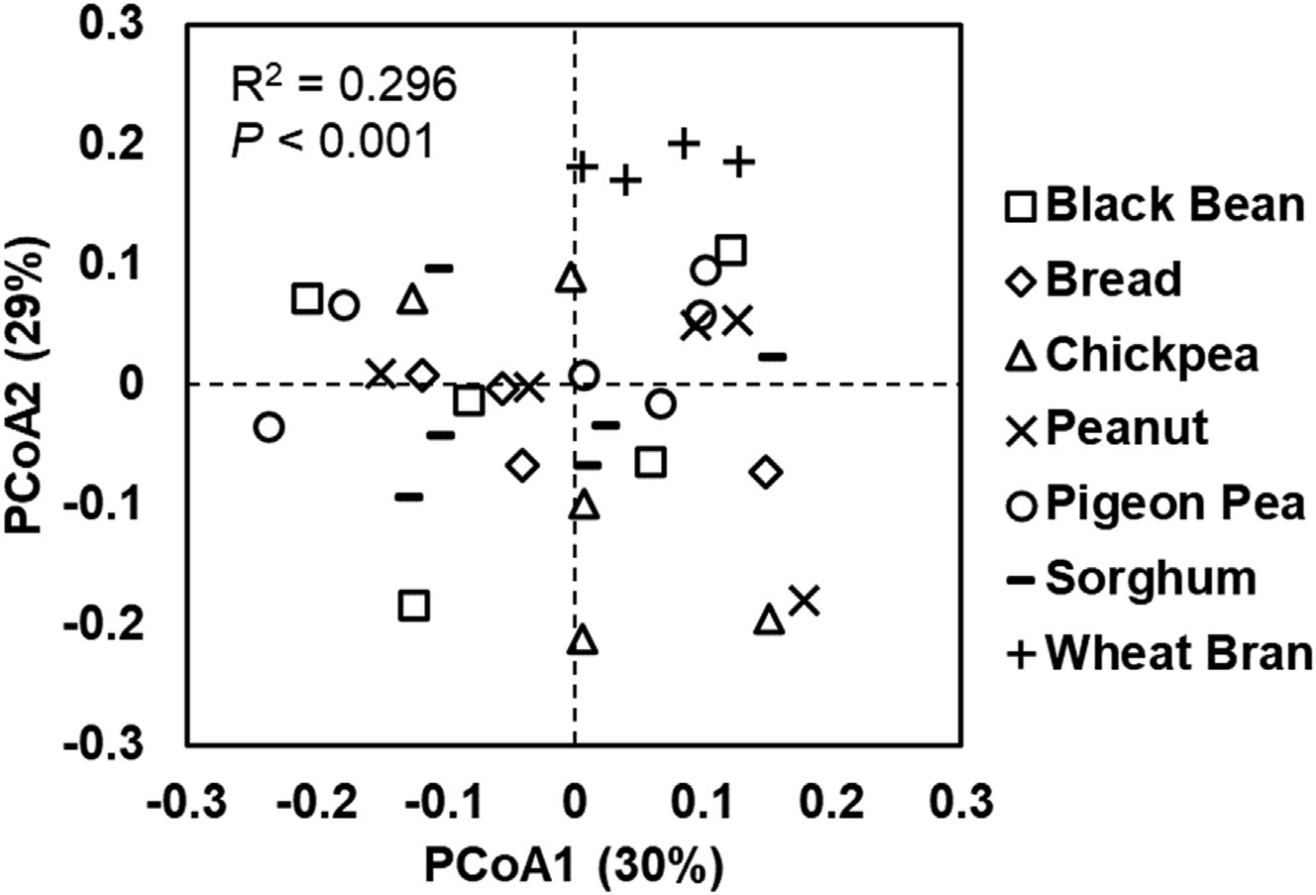
Principal coordinate analysis (PCoA) plot of Bray–Curtis dissimilarity of the ileal microbial community for growing pigs fed diets for 7 d containing different test foods. Individual symbols represent individual samples, *n* = 4–6 animals per diet. The effect of diet on the groupings was assessed using the Adonis test (999 permutations).

The α-diversity (Shannon Diversity Index) of the ileal microbial community was similar (*P* = 0.665) across all diets (Supplementary Figure S1). The Bray–Curtis dissimilarity index (β-diversity index) did not clearly separate the diets for the ileal microbial community pertaining to individual pigs (Figure 1). However, an Adonis test suggested that the diets were significantly different (*P* < 0.001). A betadisper test for intragroup variance was significant (*P* = 0.036), indicating that some differences between ileal microbial compositions across diets could be ascribed to greater intragroup variability.

### Correlations between ileal microbes and fermentation outcomes

**FIGURE 2:**
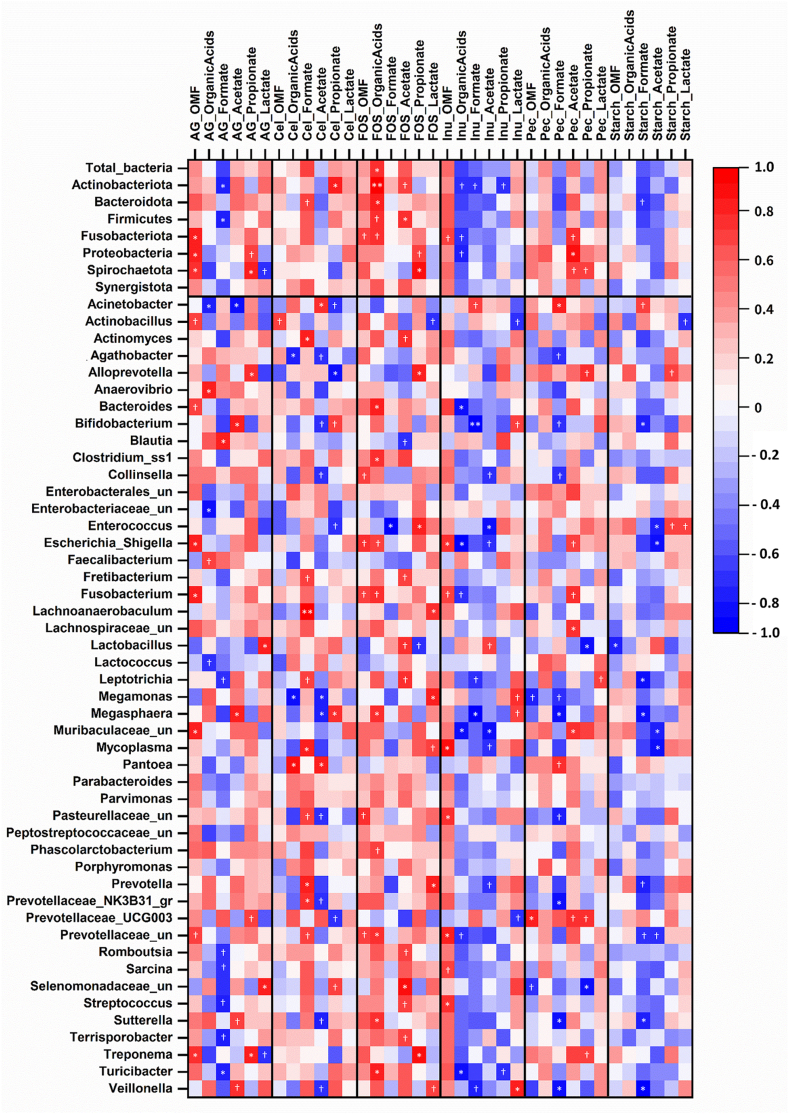
Heatmap of Pearson’s correlation coefficients between fermentation outcomes (OM fermentability and organic acid production) and the number of bacteria in the ileal inoculum per fiber substrate after in vitro fermentation. Symbols represent the *P* values: ∗∗ *P* < 0.01, ∗ *P* < 0.05, and † 0.05 < *P* < 0.10. The correlation coefficients (*r* values) and *P* values are displayed in Supplementary Table S7. AG, arabinogalactan; Cel, cellulose; FOS, fructooligosaccharides; Inu, inulin; OMF, organic matter fermentability; Pec, pectin.

Given that there were marked differences in the number of specific bacteria in the ileal digesta of the pigs, it was of interest to investigate whether the observed differences in the fermentation outcomes were correlated with differences in the bacterial taxa found in the inocula. Correlation coefficients were determined across the inocula for each fiber source between OM fermentability or organic acid production and the number of bacteria for each bacterial taxon. Fourteen statistically significant correlations (*P* ≤ 0.05; 13 positive and 1 negative) were found between OM fermentability and the concentrations of bacterial taxa found in the ileal digesta across the fiber sources. For example, *Fusobacterium* was positively correlated with the OM fermentability of AG (*r* = 0.805; *P* = 0.029) (Figure 2). In addition, 51 positive and 35 negative significant correlations (*P* ≤ 0.05) were found between organic acid production and the number of bacteria for each bacterial taxon for specific fiber sources (Figure 2). For example, *Bifidobacterium* was positively correlated with formic acid when fermenting AG (*r* = 0.816; *P* = 0.025) but negatively correlated when fermenting inulin (*r* = -0.916; *P* = 0.004) and starch (*r* = -0.775; *P* = 0.041). The *r* and *P* values of the correlations are presented in Supplementary Table S7.

## Discussion

In the present work, different diets were fed to growing pigs, ileal digesta were collected, and ileal inocula were prepared for in vitro fermentation of a wide range of fiber sources. The study showed that the ileal microbial composition, when inocula were prepared by pooling ileal digesta from pigs fed diets containing different test foods for 7 d, influenced the in vitro fermentation of fiber sources. As hypothesized, both the inoculum and fiber source influenced the in vitro ileal fermentation, resulting in differences in OM fermentability and organic acid production. However, the fiber source had a greater effect on these fermentation outcomes than the inoculum. Due to the limited amount of digesta collected per animal, a pooled inoculum was prepared per diet. Thus, the current results do not reflect variability across individual animals, but they do characterize the fermentability of different fiber sources across different ileal microbial compositions.

### In vitro OM fermentability

Feeding growing pigs with diets of varying nutrient composition (eg, 57–295 g total dietary fiber/kg diet) for 7 d resulted in different ileal microbial compositions. Dietary interventions have previously been shown to be a successful tool for changing the ileal microbial community in growing pigs using longer interventions than those applied here (22 [8], 28 [7], and 42 d [2]). However, in humans, the ileal microbial community has been shown to be affected after a 2-wk intervention [9] or even after a single meal [10,27]. The different ileal microbial community compositions obtained here through dietary intervention allowed us to interrogate our hypothesis regarding the effect of both ileal microbial community composition and fiber source on in vitro ileal fermentation.

Ileal OM fermentation was quantitatively important (3%–37%), as previously found for the in vitro fermentation of jejunal digesta [1,2] and fiber sources [11,21] using a porcine ileal inoculum. Quantitatively important ileal fermentation was also observed using a human ileal inoculum, as 29%–89% of the galactooligosaccharides and FOS were fermented after 5 h of in vitro fermentation [16]. The differences in ileal OM fermentability among fiber sources could be explained by factors such as *1*) structural differences between the fiber sources, such as monomeric composition and branching [4,16,28]. For example, in the current study, pectin that consists of different monomeric units (including galacturonic acid, rhamnose, arabinose, and galactose [29]) had a greater in vitro OM fermentability than FOS and inulin that contain only fructose monomers [30]; *2*) composition and diversity of the ileal microbial communities. This is supported by the 15 statistically significant correlations found in the current study between the number of bacteria belonging to a specific bacterial taxon and OM fermentability; *3*) fiber source preference of specific bacteria. For example, in the current study, *Escherichia-Shigella* had (or tended to have) a positive correlation with the OM fermentability of AG, FOS, and inulin.

The statistical analysis showed that ileal OM fermentability was influenced by the interaction between the fiber source and the inoculum. However, considering both the differences in *F*-values of the individual factors (930 and 58.0 for substrate and inoculum) and the higher variability between fiber sources (27% was the lowest difference in range) compared with the variability between inocula (7% was the lowest difference in range), it appears that the fiber source plays a greater role in ileal fermentation. Similarly, when using an inoculum from human ileostomates [16], both the fiber source and the donor affected the in vitro ileal fermentation, but the contribution of the fiber source appeared to be greater.

### In vitro organic acid production

In vitro organic acid production was also investigated here to measure the extent of microbial metabolism. Of the organic acids detected in this study, only formic, acetic, and lactic acids were produced in considerable amounts. In contrast, propionic and succinic acids were produced only for specific fiber sources and inocula. Formic acid was the primary fermentation product for the in vitro fermentation of AG. On average, it represented 73% of the total organic acids produced, followed by lactic and acetic acids (14% and 13%, respectively). Similar results were found when fermenting AG in vitro with a continuous culture of *Bifidobacterium longum* [31] or in batch fermentation using an ileal inoculum from pigs fed a human-type diet [11].

Based on both the *F*-values of the individual factors (eg, 489 and 13.6 for substrate and inoculum for total organic acid production) on the variability between fiber sources and inocula for organic acid production, the fiber sources appeared to play a greater role in organic acid production during in vitro ileal fermentation than the microbial composition, as described above for ileal OM fermentability. An important number of statistically significant correlations were fiber source dependent. Some of the factors described above can explain this, such as structural differences between fiber sources [28,32] and fiber source preferences of bacteria [13]. For example, *Bifidobacteria* had (or tended to have) a positive correlation with acetic acid production from AG but tended to be negatively correlate with acetic acid production from cellulose. This observation could be regarded as both involving fiber source preference and structural differences. Previous reports have shown that *Bifidobacterium longum* can utilize AG, resulting in acetic acid production in a monoculture [31], but no reports on cellulose were found. Further work is warranted to determine the cause–effect of these identified bacteria on ileal fermentation.

At first sight, there appears to be a discrepancy between the OM fermentability observations and the organic acid data. For example, AG, FOS, and inulin were all fermented similarly (about 16%), but the total organic acid production for AG was approximately 5 times higher than that for FOS and inulin. This may be partly explained in that most of the organic acid production for AG found in the current study was from formic acid, which has a relatively low molecular weight [33]. Except in the case of the peanut inoculum, the fermentation of cellulose was negligible. However, the total organic acid production was only slightly lower than that of FOS, inulin, and pectin. There is no obvious explanation for this, and it may indicate that the organic acid correction after fermentation of the blanks in this assay may not be completely accurate. The inoculum contains undigested material from the ileal digesta, providing between 35% and 50% of the total OM in the fermentation bottle. The fermentation of this material could potentially differ with and without (ie, blanks) the added fiber source. For instance, in the current study, only the peanut inoculum (blank) was fermented. Differences in total organic acid production compared with OM fermentability may also be due to differences in the production of metabolites and gases [34] that were not determined here. These unexplained discrepancies mean that the organic acid data should be interpreted cautiously. Overall, however, they provide corroborative evidence for the effect of dietary composition on in vitro ileal fermentation.

In conclusion, in vitro ileal fermentation of different well-characterized fiber sources was quantitatively important when using the growing pig as an animal model for the adult humans. There was a statistically significant effect of the dietary composition on fermentation. Both the fiber source and the inoculum (bacterial composition) influenced the in vitro OM fermentability and the organic acids produced. However, based on the source of variation, the fiber source made a greater contribution to ileal fermentation. However, based on several observed statistically significant correlations, the contribution of the fiber source to fermentation appeared to be modulated by the number of specific bacteria. For example, the number of *Prevotellaceae* UCG003 was related to the in vitro ileal fermentation of pectin only. These results suggest that dietary intervention could play an important role in influencing ileal fermentation by either providing specific substrates or by shaping the ileal microbial composition to promote the production of specific organic acids.

## Author contributions

The authors’ responsibilities were as follows—SMH, PJM: designed the in vivo study; CAM, PJM, AMEH, WCM: designed the in vitro study; SMH, NS, WW, AMEH: were responsible for the in vivo study; AMEH, WW: conducted the in vitro fermentation study; CAM, WW, AMEH: analyzed the samples; EAR, AMEH: performed the bioinformatics; AMEH: performed the statistical analysis (with the assistance of CAM) and wrote the manuscript, which was critically reviewed by CAM and PJM; and all authors have read and approved the final manuscript.

## Funding

Supported by the Centre of Research Excellence Fund from the Tertiary Education Commission and the New Zealand Ministry of Education.

## Author disclosures

The authors declare no conflict of interest.
