# Supplementary material for: In Vitro Ileal Fermentation is Affected More by the Fiber Source Fermented than the Ileal Microbial Composition in Growing Pigs
Source: Curr Dev Nutr. 2023 Apr 11;7(5):100076. doi: 10.1016/j.cdnut.2023.100076 (PMC10172865; doi:10.1016/j.cdnut.2023.100076)
Supplement: Multimedia component 1 [file mmc1.docx]

# Supplementary Data

## Supplementary methods

### Ingredients and diet preparations

All test foods were food-grade and sourced commercially, except the wheat bread. Pigeon peas, black beans, chickpeas, and sorghum were prepared at the Food Pilot Plant (Massey University) as described below, stored at -20°C, and defrosted before use. The non-protein-containing ingredients were mixed in the Feed Mill (Massey University) and stored at -20°C until use. The mix of non-protein-containing ingredients was specific for each diet (Supplementary Table 1). In addition, two indigestible markers, titanium dioxide (TiO_2_; 4 g/kg DM) and celite (7.5 g/kg DM), were added to each diet, but they were not considered in this study. On days 6 and 7, the sorghum was prepared fresh before feeding. The wheat bran was prepared fresh for all meals.

*Black Bean*. Dried black beans were soaked in excess water (3:4 w/v ratio dry beans to water) at room temperature for 18 h (29). Black beans were drained. Table salt (720 mg per 100 g of soaked black beans) and water (1:1.15 w/v ratio of soaked black beans to water) was added before cooking in a commercial pressure retort (Mauri Engineering, Palmerston North, NZ) at 100 kPa and 121°C for 20 min.

*Bread*. The bread was baked at the Department of Food Science and Human Nutrition Pilot Processing Plant at the University of Illinois Urbana-Champaign, according to a standardized recipe. First, dry yeast and sucrose were combined with warm water. After it started foaming (i.e., yeast was active), salt, butter, flour, and the indigestible makers (titanium dioxide and celite) were added. Next, a commercial mixer (Hobart Legacy Mixer, Troy, OH) was used to knead the bread dough. Then the dough was portioned into baking trays (23 x 13 x 6 cm) and allowed to rest for one hour. The bread was baked at 175°C for 35 minutes. The loaves of bread were then cooled to room temperature and frozen. Before feeding, bread was thawed, sliced (13 mm slices), lightly toasted, and diced.

*Chickpeas*. Canned chickpeas (Sofia, Davis Food Ingredients) were drained and processed in a food processor for eight pulses over 15 seconds or until they reached a “chewed-like” texture.

*Peanuts.* Peanuts were sourced de-shelled and roasted. Roasted peanuts were coarsely ground.

*Pigeon peas*. Dried pigeon peas were prepared as described for the black beans, but with a cooking time of 10 min (29).

*Sorghum. S*orghum flour was prepared as a porridge with a final ratio of 1:4 flour to water (w/v). The sorghum flour was mixed with half the water before adding to boiling water (i.e., the other half of water). It simmered for 20 min while constantly stirring until it acquired a thick, porridge-like consistency (30).

*Wheat bran*. Wheat bran (Kellogg’s® All-Bran®) was mixed with water until the indigestible markers were homogeneously distributed.

### Microbial analysis

The total number of bacteria (i.e., 16S rRNA gene copies) was determined using a quantitative PCR (qPCR). A qPCR instrument (LightCycler 480, Roche) was used to determine the total concentration of 16S rRNA gene copies in ileal digesta in duplicate (1). SyBr Green detection chemistry (Roche) was used to amplify the DNA extracted for the ileal samples and the standard DNA (*Escherichia coli,* Nissle). The forward primer used was 5’-TCCTACGGGAGGCAGCAGT, and the reverse primer was (5’-GGACTACCAGGGTATCTAATCCTGTT. Every qPCR run included an activation cycle (95°C, 5 min), 40 run cycles (i.e., denaturation (95°C, 30 s), annealing 60°C, 60 s) and extension (72°C, 60 s)) and one melting curve (60–95°C at 0·1°C/s). Data output was analyzed using the LightCycler 480 Software (Version 1.5, Roche). The total number of 16S rRNA gene copies in the ileal digesta and the relative abundance of the taxa were used to determine the number of 16S rRNA gene copies per taxa with the assumption that each taxon has an equal number of 16s rRNA gene copies (calculation in Supplementary Methods).

Extracted DNA samples were sent to the Massey Genome Service (Massey University) for Illumina MiSeq sequencing to determine the taxonomic composition (1). To amplify the V3-V4 hypervariable region of the 16S rRNA gene, the following primers were used: 16SF_V3 (5’ - AATGATACGGCGACCACCGAGATCTACAC-index-TATGGTAATTGGCCTACGGGAGGCAGCAG) and 16SR_V4 (5’ -CAAGCAGAAGACGGCATACGAGAT-index-AGTCAGTCAGCCGGACTACHVGGGTWTCTAAT). 16SF_V3 (5’ - AATGATACGGCGACCACCGAGATCTACAC-index-TATGGTAATTGGCCTACGGGAGGCAGCAG) and 16SR_V4 (5’ -CAAGCAGAAGACGGCATACGAGAT-index-AGTCAGTCAGCCGGACTACHVGGGTWTCTAAT). The 96 libraries were prepared using the Illumina 16S V3-V4 rRNA library preparation method. The Massey Genome Service has dual index PCR primers which flank the V3-V4 hyper-variable region of 16S rRNA, which uses a Single Step PCR Library preparation method to prepare the libraries. The libraries were run on an Illumina MiSeq™ 2X 250 base PE, version 2 chemistry. In some samples, the quality or concentration of the extracted DNA or both was too low to perform qPCR analysis and Illumina sequencing. Thus, the sample size per treatment differs.

The bioinformatic analysis was done using Mothur V1.44.2 (2, 3). Briefly, a total of 3,319,622 paired-end reads were detected. These reads were assembled and underwent quality control removing all reads with more than eight homopolymers and uncalled base. The average length of the sequences was 420 bp. Both the SLIVA database (version 138) (4) and the Greengenes database (version 13_8) (5) were used to align the sequences. After alignment, sequences were pre-clustered (4 bp) to remove noise and reduce the effect of sequencing errors. Chimeras were removed using VSEARCH (6), and all non-bacterial sequences were excluded. The remaining 2,640,909 reads were clustered into OTUs with a 97% cutoff. A subsample of 47,642 reads per sample was used. The BIOM table generated after alignment with the SILVA database was used as input for Calypso (version 8.84) (7) to obtain the taxonomic composition, Shannon Diversity Index numbers and the principal coordinates analysis (PCoA) with the Bray-Curtis dissimilarities of the ileal microbiota. Phylogenetic Investigation of Communities by Reconstruction of Unobserved States (PICRUSt) (8) required the BIOM table generated after alignment with the Greengenese database to predict the metabolic activity of the ileal microbiota.

### Calculations

The following equations were used to determine *in vitro* ileal OM fermentability (21):

1. OM fermentability (%) = (OM _before fermentation_ – [OM _after fermentation_ – ((OM _blank initial_ + OM _blank final_)/2)]) / OM _before fermentation_ x 100

where OM _blank initial_ and OM _blank final_ are the amounts of OM in the blanks before (initial) and after (final) *in vitro* fermentation, respectively. For each diet, the *in vitro* ileal fermentation had its own blanks.

The *in vitro* ileal production of organic acids was determined as described previously (21), using the following equations:

1. Organic acid production (mmol/kg substrate DM incubated) = (organic acid _after fermentation_ (mmol) – [(organic acid _blank initial_ + organic acid _blank final_)/2]) / kg DM substrate

where organic acid _blank initial_ and organic acid _blank final_ are the organic acids (mmol) in the blanks before (initial) and after (final) *in vitro* fermentation, respectively. The *in vitro* ileal fermentation had its own blanks for each inoculum.

To determine the number of bacteria per taxa in ileal digesta, it was assumed that each taxon had an equal number of 16S rRNA gene copies. The number of bacteria per taxa in ileal digesta was calculated as follows (1):

1. Number of bacteria per taxa_ileal digesta_ (16S rRNA gene copy number/g wet digesta) = total number of bacteria_ileal digesta_ (16S rRNA gene copy number/g wet digesta) x relative abundance _taxa_ (%) / 100

## Supplementary Tables

**Supplementary Table 1:** The ingredient and determined nutrient composition of the diets

|  | |  | Black bean | Bread | Chickpea | Peanut | Pigeon pea | Sorghum | Wheat bran |
| --- | --- | --- | --- | --- | --- | --- | --- | --- | --- |
| Ingredient, g/kg DM^1^ | | | | | | | | | |
|  | Test food | | 448 | 946 | 522 | 357 | 488 | 942 | 803 |
|  | Maize starch | | 314 | - | 244 | 405 | 274 | - | - |
|  | Cellulose | | 30 | - | 30 | 30 | 30 | - | 71 |
|  | Rapeseed oil | | 50 | - | 50 | 50 | 50 | - | 71 |
|  | Sucrose | | 100 | - | 100 | 100 | 100 | - | - |
|  | Vitamin/mineral mix^2^ | | 1.5 | 1.5 | 1.5 | 1.5 | 1.5 | 1.5 | 1.5 |
|  | Dicalcium phosphate | | 25 | 25 | 25 | 25 | 25 | 25 | 25 |
|  | Calcium bicarbonate | | 3 | 3 | 3 | 3 | 3 | 3 | 3 |
|  | Potassium bicarbonate | | 10 | 10 | 10 | 10 | 10 | 10 | 10 |
|  | Sodium bicarbonate | | 3 | 3 | 3 | 3 | 3 | 3 | 3 |
|  | Sodium chloride | | 4 | - | - | 4 | 4 | 4 | 1 |
|  | Titanium dioxide | | 4 | 4 | 4 | 4 | 4 | 4 | 4 |
|  | Celite | | 7.5 | 7.5 | 7.5 | 7.5 | 7.5 | 7.5 | 7.5 |
|  |  | |  |  |  |  |  |  |  |
| Nutrient, g/kg DM^1^ | | | | | | | | | |
|  | Ash | | 65.6 | 79.3 | 65.6 | 54.2 | 62.8 | 62.9 | 75.1 |
|  | Crude protein | | 112 | 114 | 110 | 113 | 131 | 95 | 110 |
|  | Total lipids | | 78.0 | 82.1 | 113 | 265 | 79.5 | 49.8 | 123 |
|  | Starch | | 431 | 535 | 440 | 396 | 436 | 648 | 230 |
|  | Total dietary fiber | | 176 | 57.2 | 129 | 57.0 | 144 | 72.6 | 295 |
|  | - Insoluble fiber | | 146 | 46.8 | 121 | 53.4 | 140 | 67.7 | 278 |
|  | - Soluble fiber | | 30 | 11.4 | 8.8 | 4.15 | 5.23 | 4.87 | 16.0 |
|  | Insoluble:soluble fiber ratio | | 4.90 | 4.09 | 13.8 | 12.9 | 26.8 | 13.9 | 17.4 |
|  | Gross energy MJ/kg | | 17.4 | 16.9 | 18.0 | 20.9 | 17.1 | 16.3 | 18.3 |
| ^1^ DM, dry matter  ^2^ The vitamin and mineral premix supplied (per kg DM diet): Ca, 230 mg; Cu, 5.1 ppm; I, 0.6 ppm; Fe, 62 ppm; Mn, 30 ppm; Se, 0.15 ppm; Zn, 50 ppm; niacin, 22 mg; cobalamin, 0.02 μg; pantothenic acid, 12 mg; riboflavin, 3.3 mg; menadione, 0.7 mg; biotin, 0.2 mg; retinyl acetate, 1.7 μg; cholecalciferol, 0.03 μg; tocopheryl acetate, 32 μg; pyridoxine, 0.12 mg; folate, 0.79 mg; thiamin, 0.12 mg. | | | | | | | | | |

**Supplementary Table 2:** Overview of the ileal digesta samples collected during the experimental periods with the allocated diet for that pig^1^

| Period | Pig 1 | Pig 2 | Pig 3 | Pig 4 | Pig 5 | Pig 6 | Pig 7 | Pig 8 | Pig 9 | Pig 10 | Pig 11 | Pig 12 | Pig 13 |
| --- | --- | --- | --- | --- | --- | --- | --- | --- | --- | --- | --- | --- | --- |
| 1 | - | - | PP | BB | WB | - | - | S | B | - | P | CP | - |
| 2 | - | PP | - | WB | - | - | S | B | P | - | CP | - | - |
| 3 | - | - | - | PP | BB | S | WB | - | - | - | B | P | CP |
| 4 | Protein-free diet | | | | | | | | | | | | |
| 5 | Basal diet | | | | | | | | | | | | |
| 6 | PP | BB | WB | - | S | B | P | CP | - | - | - | - | - |
| 7 | S | - | - | - | PP | BB | CP | WB | - | B | - | - | P |
| 8 | BB | WB | - | S | B | P | - | PP | - | CP | - | - | BB |
| 9 | - | S | - | - | - | PP | - | P | - | - | - | - | - |
| ^1^A line represents no digesta sample collected from that pig during that period. B, bread; BB, black beans; CP, chickpeas; P, peanuts; PP, pigeon peas; S, sorghum; WB, wheat bran | | | | | | | | | | | | | |

**Supplementary Table 3:** Production of propionic and succinic acids during *in vitro* fermentation of dietary fiber substrates using pooled ileal inocula from growing pigs fed diets containing different test foods^1^

| Organic acid | Inoculum | Substrate | | | | | | | |
| --- | --- | --- | --- | --- | --- | --- | --- | --- | --- |
|  |  | AG | Cellulose | FOS | Inulin | Pectin | High-amylose starch |  | Mean  (range) |
| *mmol/kg DM substrate* | | | | | | | | | |
| Propionic acid | Black bean | 0.376 ± 0.235^b^ | ND | 2.92 ± 0.263^a^ | 0.343 ± 0.304^b,§^ | 3.83 ± 0.304^a,†^ | 2.24 ± 0.263^a,‡^ |  | 1.64  (0.00 - 5.12) |
|  | Bread | 3.04 ± 0.946 | 1.24 ± 1.09^†‡^ | 3.34 ± 1.09 | 4.44 ± 0.846^‡^ | ND | 6.94 ± 1.34^†‡^ |  | 3.17  (0.00 - 6.96) |
|  | Chickpea | ND | 4.66 ± 0.581^†^ | ND | ND | ND | ND |  | 0.777  (0.00 - 6.20) |
|  | Peanut | 2.32 ± 0.465 | 1.68 ± 0.465^†‡^ | 2.25 ± 0.465 | ND | 3.40 ± 0.416^†^ | 1.91 ± 0.465^‡§^ |  | 1.93  (0.00 - 5.44) |
|  | Pigeon pea | ND | 1.46 ± 0.156^‡^ | 1.59 ± 0.180 | ND | ND | 0.855 ± 0.156^§^ |  | 0.737  (0.00 - 2.14) |
|  | Sorghum | ND | ND | ND | ND | 0.813 ± 0.134^‡^ | ND |  | 0.286  (0.00 - 1.65) |
|  | Wheat bran | ND | ND | ND | 8.49 ± 0.272^†^ | ND | 6.72 ± 0.352^†^ |  | 2.65  (0.00 - 9.87) |
|  |  |  |  |  |  |  |  |  |  |
|  | Mean  (range) | 0.894 ± 0.169  (0.00 - 5.24) | 1.36 ± 0.188  (0.00 - 6.20) | 1.49 ± 0.187  (0.00 - 5.10) | 1.95 ± 0.157  (0.00 - 9.87) | 1.20 ± 0.188  (0.00 - 5.44) | 2.69 ± 0.219  (0.00 - 7.60) |  |  |
|  |  |  |  |  |  |  |  |  |  |
| Succinic acid | Black bean | ND | ND | ND | ND | ND | ND |  |  |
|  | Bread | 5.67 ± 0.533^†^ | ND | ND | ND | ND | ND |  |  |
|  | Chickpea | 6.31 ± 0.477^†^ | ND | ND | ND | ND | ND |  |  |
|  | Peanut | 5.99 ± 0.477^†^ | ND | ND | ND | ND | ND |  |  |
|  | Pigeon pea | 1.50 ± 0.533^‡^ | ND | ND | ND | ND | ND |  |  |
|  | Sorghum | 0.23 ± 0.533^‡^ | ND | ND | ND | ND | ND |  |  |
|  | Wheat bran | 2.23 ± 0.447^‡^ | ND | ND | ND | ND | ND |  |  |
|  |  |  |  |  |  |  |  |  |  |
| *P* value |  | Inoculum (I) | Substrate (S) | I x S |  |  |  |  |  |
|  | Propionic acid | <0.001 | <0.001 | <0.001 |  |  |  |  |  |
|  | Succinic acid | <0.001 | - | - |  |  |  |  |  |
| ^1^ Values are means ± SEM, *n* = 5 fermentation bottles. A two-way ANOVA model was used to assess the effect of inoculum, substrate, and their interaction for propionic acid. A different repeated statement was required to have similar studentized residuals as described in the statistical analysis section. Means in a row (i.e., inoculum effect) with different letters differ (*P* ≤ 0.05), and means in a column (i.e., substrate effect) with different symbols differ (*P* ≤ 0.05). AG, arabinogalactan; DM, dry matter; FOS, fructooligosaccharides; ND, not detected. | | | | | | | | | |

**Supplementary Table 4:** Frequency of occurrence of taxa in ileal digesta from growing pigs fed diets for seven days containing different test foods^1^

|  |  | Diet | | | | | | |  |  |  |
| --- | --- | --- | --- | --- | --- | --- | --- | --- | --- | --- | --- |
| Phylum | Genera | Black bean | Bread | Chickpea | Peanut | Pigeon pea | Sorghum | Wheat bran | SEM |  | *P* value |
| Sample size, *n*^2^ | | 4 | 5 | 4 | 5 | 6 | 6 | 6 |  |  |  |
| Actinobacteria | | 1.00 | 1.00 | 1.00 | 1.00 | 1.00 | 1.00 | 1.00 | - |  | - |
|  | *Actinomyces* | 1.00 | 1.00 | 1.00 | 1.00 | 1.00 | 1.00 | 1.00 | - |  | - |
|  | *Bifidobacterium* | 1.00^a^ | 0.500^b^ | 1.00^a^ | 0.600^ab^ | 0.857^ab^ | 0.500^b^ | 1.00^a^ | 0.024 |  | 0.050 |
|  | *Collinsella* | 0.800^ab^ | 1.00^a^ | 0.600^ab^ | 1.00^a^ | 1.00^a^ | 0.500^b^ | 1.00^a^ | 0.017 |  | 0.050 |
| Bacteroidetes | | 1.00 | 1.00 | 1.00 | 1.00 | 1.00 | 1.00 | 1.00 | - |  | - |
|  | *Alloprevotella* | 1.00 | 1.00 | 0.800 | 1.00 | 1.00 | 1.00 | 1.00 | 0.005 |  | 0.306 |
|  | *Bacteroides* | 1.00 | 1.00 | 1.00 | 1.00 | 1.00 | 1.00 | 1.00 | - |  | - |
|  | *Muribaculaceae_unclassified* | 1.00 | 0.750 | 0.600 | 0.800 | 0.857 | 1.00 | 0.833 | 0.024 |  | 0.118 |
|  | *Parabacteroides* | 1.00 | 1.00 | 1.00 | 1.00 | 1.00 | 1.00 | 1.00 | - |  | - |
|  | *Porphyromonas* | 1.00 | 1.00 | 1.00 | 1.00 | 1.00 | 1.00 | 1.00 | - |  | - |
|  | *Prevotella* | 1.00 | 1.00 | 1.00 | 1.00 | 1.00 | 1.00 | 1.00 | - |  | - |
|  | *Prevotellaceae_NK3B31_group* | 1.00 | 1.00 | 1.00 | 1.00 | 1.00 | 1.00 | 1.00 | - |  | - |
|  | *Prevotellaceae_UCG003* | 1.00 | 1.00 | 0.800 | 1.00 | 1.00 | 1.00 | 1.00 | 0.005 |  | 0.306 |
|  | *Prevotellaceae_unclassified* | 1.00 | 1.00 | 1.00 | 1.00 | 1.00 | 1.00 | 1.00 | - |  | - |
| Firmicutes | | 1.00 | 1.00 | 1.00 | 1.00 | 1.00 | 1.00 | 1.00 | - |  | - |
|  | *Agathobacter* | 1.00 | 1.00 | 1.00 | 0.800 | 0.571 | 0.667 | 1.00 | 0.015 |  | 0.062 |
|  | *Anaerovibrio* | 0.600^ab^ | 1.00^a^ | 1.00^a^ | 1.00^a^ | 0.857^ab^ | 0.500^b^ | 1.00^a^ | 0.015 |  | 0.050 |
|  | *Blautia* | 1.00 | 1.00 | 0.800 | 1.00 | 0.857 | 1.00 | 1.00 | 0.007 |  | 0.153 |
|  | *Cellulosilyticum* | 0.800 | 0.500 | 0.800 | 0.800 | 0.714 | 0.667 | 0.750 | 0.039 |  | 0.367 |
|  | *Clostridium_sensu_stricto_1* | 1.00 | 1.00 | 1.00 | 1.00 | 1.00 | 1.00 | 1.00 | - |  | - |
|  | *Enterococcus* | 1.00 | 1.00 | 1.00 | 1.00 | 1.00 | 1.00 | 1.00 | - |  | - |
|  | *Faecalibacterium* | 0.600^ab^ | 0.500^b^ | 0.800^ab^ | 0.600^ab^ | 0.571^ab^ | 0.500 | 1.00^a^ | 0.038 |  | 0.050 |
|  | *Fusicatenibacter* | 0.600^ab^ | 0.250^bc^ | 0.200^bc^ | 0.000^c^ | 0.286^bc^ | 0.167^bc^ | 1.00^a^ | 0.026 |  | 0.006 |
|  | *Lachnoanaerobaculum* | 1.00 | 1.00 | 1.00 | 1.00 | 1.00 | 1.00 | 1.00 | - |  | - |
|  | *Lachnospiraceae_unclassified* | 1.00 | 1.00 | 1.00 | 1.00 | 1.00 | 1.00 | 1.00 | - |  | - |
|  | *Lactobacillus* | 1.00 | 1.00 | 1.00 | 1.00 | 1.00 | 1.00 | 1.00 | - |  | - |
|  | *Lactococcus* | 1.00 | 0.750 | 0.800 | 0.600 | 1.00 | 1.00 | 1.00 | 0.018 |  | 0.118 |
|  | *Leuconostoc* | 0.600^ab^ | 0.250^bc^ | 0.200^bc^ | 0.200^bc^ | 0.714^ab^ | 0.833^a^ | 0.000^c^ | 0.030 |  | 0.036 |
|  | *Megamonas* | 1.00^a^ | 0.750^ab^ | 1.00^a^ | 0.400^b^ | 0.857^ab^ | 0.333^b^ | 1.00^a^ | 0.021 |  | 0.013 |
|  | *Megasphaera* | 1.00^a^ | 1.00^a^ | 1.00^a^ | 1.00^a^ | 1.00^a^ | 0.333^b^ | 1.00^a^ | 0.005 |  | 0.013 |
|  | *Mycoplasma* | 1.00 | 1.00 | 0.800 | 0.600 | 0.857 | 0.500 | 1.00 | 0.020 |  | 0.116 |
|  | *Parvimonas* | 1.00 | 1.00 | 1.00 | 1.00 | 1.00 | 1.00 | 1.00 | - |  | - |
|  | *Phascolarctobacterium* | 1.00 | 1.00 | 1.00 | 1.00 | 1.00 | 1.00 | 1.00 | - |  | - |
|  | *Romboutsia* | 1.00 | 1.00 | 1.00 | 1.00 | 1.00 | 1.00 | 1.00 | - |  | - |
|  | *Sarcina* | 1.00 | 1.00 | 1.00 | 0.800 | 1.00 | 1.00 | 1.00 | 0.005 |  | 0.306 |
|  | *Selenomonadaceae_unclassified* | 1.00 | 1.00 | 1.00 | 1.00 | 1.00 | 0.833 | 1.00 | 0.003 |  | 0.315 |
|  | *Sharpea* | 0.200 | 0.000 | 0.200 | 0.200 | 0.429 | 0.000 | 0.00 | 0.019 |  | 0.062 |
|  | *Streptococcus* | 1.00 | 1.00 | 1.00 | 1.00 | 1.00 | 1.00 | 1.00 | - |  | - |
|  | *Terrisporobacter* | 1.00 | 1.00 | 1.00 | 1.00 | 1.00 | 1.00 | 1.00 | - |  | - |
|  | *Turicibacter* | 1.00 | 1.00 | 1.00 | 1.00 | 1.00 | 1.00 | 1.00 | - |  | - |
|  | *Veillonella* | 1.00 | 1.00 | 1.00 | 1.00 | 1.00 | 1.00 | 1.00 | - |  | - |
|  | *Weissella* | 0.800^ab^ | 0.500^bc^ | 0.400^bc^ | 0.200^c^ | 0.857^ab^ | 1.00^a^ | 1.00^a^ | 0.027 |  | 0.004 |
| Fusobacteria | | 1.00 | 1.00 | 1.00 | 1.00 | 1.00 | 1.00 | 1.00 | - |  | - |
|  | *Fusobacterium* | 1.00 | 1.00 | 1.00 | 1.00 | 1.00 | 1.00 | 1.00 | - |  | - |
|  | *Leptotrichia* | 1.00 | 1.00 | 0.800 | 1.00 | 1.00 | 0.667 | 0.75 | 0.017 |  | 0.134 |
| Proteobacteria | | 1.00 | 1.00 | 1.00 | 1.00 | 1.00 | 1.00 | 1.00 | - |  | - |
|  | *Acinetobacter* | 1.00^a^ | 1.00^a^ | 0.800^ab^ | 0.600^ab^ | 0.571^b^ | 1.00^a^ | 1.00^a^ | 0.016 |  | 0.031 |
|  | *Actinobacillus* | 1.00 | 1.00 | 1.00 | 1.00 | 1.00 | 1.00 | 1.00 | - |  | - |
|  | *Enterobacterales_unclassified* | 1.00 | 1.00 | 1.00 | 1.00 | 1.00 | 1.00 | 1.00 | - |  | - |
|  | *Enterobacteriaceae_unclassified* | 1.00 | 1.00 | 0.800 | 1.00 | 1.00 | 1.00 | 1.00 | 0.005 |  | 0.306 |
|  | *Erwiniaceae_unclassified* | 0.200 | 0.000 | 0.400 | 0.000 | 0.143 | 0.833 | 0.00 | 0.017 |  | 0.002 |
|  | *Escherichia-Shigella* | 1.00 | 1.00 | 1.00 | 1.00 | 1.00 | 1.00 | 1.00 | - |  | - |
|  | *Kosakonia* | 0.600^ab^ | 0.500^abc^ | 0.000^c^ | 0.200^bc^ | 0.714^ab^ | 1.00^a^ | 0.250^bc^ | 0.031 |  | 0.013 |
|  | *Pantoea* | 0.600 | 0.250 | 0.600 | 0.400 | 0.714 | 0.833 | 0.50 | 0.044 |  | 0.070 |
|  | *Pasteurellaceae_unclassified* | 1.00 | 1.00 | 1.00 | 1.00 | 1.00 | 1.00 | 1.00 | - |  | - |
|  | *Peptostreptococcaceae_unclassified* | 1.00 | 1.00 | 1.00 | 1.00 | 1.00 | 1.00 | 1.00 | - |  | - |
|  | *Pseudomonas* | 0.200^cd^ | 0.500^abc^ | 0.000^c^ | 0.400^bc^ | 0.714^ab^ | 1.00^a^ | 0.250^c^ | 0.031 |  | 0.034 |
|  | *Rahnella1* | 0.200^b^ | 0.000^b^ | 0.000^b^ | 0.200^b^ | 0.429^ab^ | 0.833^a^ | 0.000^b^ | 0.017 |  | 0.002 |
|  | *Sutterella* | 1.00 | 1.00 | 1.00 | 1.00 | 1.00 | 1.00 | 1.00 | - |  | - |
|  | *Yersiniaceae_unclassified* | 0.200^ab^ | 0.000^b^ | 0.000^b^ | 0.000^b^ | 0.286^ab^ | 0.667^a^ | 0.000^b^ | 0.014 |  | 0.013 |
| Spirochaetes | | 0.800 | 0.750 | 0.800 | 0.600 | 0.571 | 0.667 | 1.00 | 0.033 |  | 0.062 |
|  | *Treponema* | 0.800 | 0.750 | 0.800 | 0.600 | 0.571 | 0.667 | 1.00 | 0.033 |  | 0.062 |
| Synergistetes | | 1.00 | 1.00 | 1.00 | 1.00 | 1.00 | 1.00 | 0.750 | 0.007 |  | 0.292 |
|  | *Fretibacterium* | 1.00 | 1.00 | 1.00 | 1.00 | 1.00 | 1.00 | 0.750 | 0.007 |  | 0.292 |
| ^1^ Values are frequencies with pooled SEM, *n* = 4-6 animals per diet. A value of 1 represents 100% frequency, and 0 represents 0% frequency. Only taxa with >1% relative abundance in at least one sample were considered. The frequency analysis was performed using a binary logistic regression with 0 when the taxon was absent and 1 when the taxon was present. No statistical analysis was performed if the frequency was 1 for all diets. Frequencies in a row with a different letter differ (*P* ≤ 0.05).  ^2^ *n* indicates the number of replicates. The different number of replicates resulted from either removing one pig that displayed coprophagy or the extracted DNA having low quality or concentration for 16S rRNA gene sequencing. | | | | | | | | | | | |

**Supplementary Table 5:** Effect of diet on the number of bacteria in ileal digesta from growing pigs fed diets containing different test foods^1^

|  |  | Diet | | | | | | |  |  |  |
| --- | --- | --- | --- | --- | --- | --- | --- | --- | --- | --- | --- |
| Phylum/Genus | | Black bean | Bread | Chickpea | Peanut | Pigeon pea | Sorghum | Wheat bran | SEM |  | *P* value |
| Sample size, *n*^2^ | | 4 | 5 | 4 | 5 | 6 | 6 | 6 |  |  |  |
|  | | *Log_10_ 16S rRNA gene copies/g wet digesta* | | | | | | |  |  |  |
| Total bacteria | | 10.6^ab^ | 11.1^a^ | 10.8^ab^ | 10.9^ab^ | 11.1^ab^ | 10.6^b^ | 10.3^b^ | 0.180 |  | 0.002 |
| Actinobacteria | | 8.42 | 8.73 | 8.98 | 8.83 | 8.82 | 8.23 | 8.05 | 0.308 |  | 0.308 |
|  | *Actinomyces* | 7.67^ab^ | 8.32^ab^ | 7.95^ab^ | 7.82^ab^ | 8.30^a^ | 7.66^ab^ | 7.43^b^ | 0.192 |  | 0.036 |
|  | *Bifidobacterium* | 7.73^a^ | 6.77^ab^ | 8.29^ab^ | 7.34^ab^ | 7.71^ab^ | 5.98^b^ | 6.87^ab^ | 0.556 |  | 0.009 |
|  | *Collinsella* | 6.99^ab^ | 7.61^a^ | 6.68^ab^ | 7.74^ab^ | 7.11^ab^ | 6.04^b^ | 7.47^a^ | 0.357 |  | 0.010 |
| Bacteroidetes | | 9.79^b^ | 10.3^a^ | 9.87^ab^ | 10.3^ab^ | 10.2^ab^ | 9.59^ab^ | 9.83^ab^ | 0.177 |  | 0.021 |
|  | *Alloprevotella* | 7.95 | 7.82 | 7.12 | 7.89 | 8.15 | 7.95 | 7.90 | 0.430 |  | 0.710 |
|  | *Bacteroides* | 9.29^ab^ | 9.79^a^ | 9.33^ab^ | 9.81^ab^ | 9.67^ab^ | 9.17^ab^ | 8.64^b^ | 0.229 |  | 0.038 |
|  | *Muribaculaceae_unclassified* | 7.01 | 6.71 | 6.40 | 7.34 | 7.02 | 6.40 | 6.58 | 0.417 |  | 0.606 |
|  | *Parabacteroides* | 7.47^b^ | 8.23^a^ | 7.50^abc^ | 7.79^abc^ | 7.92^abc^ | 7.89^ab^ | 6.73^c^ | 0.252 |  | 0.001 |
|  | *Porphyromonas* | 7.82 | 8.58 | 7.85 | 8.04 | 8.89 | 8.21 | 7.32 | 0.491 |  | 0.351 |
|  | *Prevotella* | 9.29^ab^ | 9.81^a^ | 9.29^ab^ | 9.45^ab^ | 9.89^ab^ | 8.93^b^ | 9.71^a^ | 0.205 |  | 0.009 |
|  | *Prevotellaceae_NK3B31_group* | 7.61 | 8.22 | 7.81 | 7.97 | 7.89 | 7.33 | 7.90 | 0.490 |  | 0.908 |
|  | *Prevotellaceae_UCG003* | 7.82 | 7.79 | 7.43 | 8.09 | 7.95 | 8.15 | 7.88 | 0.370 |  | 0.851 |
|  | *Prevotellaceae_unclassified* | 8.85 | 9.30 | 8.72 | 9.17 | 9.16 | 8.52 | 8.52 | 0.241 |  | 0.139 |
| Firmicutes | | 10.3^ab^ | 10.9^a^ | 10.6^ab^ | 10.5^ab^ | 10.8^ab^ | 10.2^b^ | 9.85^b^ | 0.200 |  | 0.005 |
|  | *Agathobacter* | 7.15^ab^ | 7.52^ab^ | 6.93^ab^ | 7.28^ab^ | 6.54^ab^ | 6.14^b^ | 7.86^a^ | 0.352 |  | 0.027 |
|  | *Anaerovibrio* | 6.66^bc^ | 7.40^b^ | 7.39^ab^ | 8.09^abc^ | 7.10^abc^ | 5.74^c^ | 8.82^a^ | 0.398 |  | <0.001 |
|  | *Blautia* | 7.25^b^ | 7.44^ab^ | 7.17^ab^ | 7.81^ab^ | 7.45^ab^ | 7.18^b^ | 8.47^a^ | 0.329 |  | 0.008 |
|  | *Cellulosilyticum* | 7.79 | 7.51 | 6.48 | 8.34 | 7.08 | 7.06 | 6.27 | 0.484 |  | 0.068 |
|  | *Clostridium_sensu_stricto_1* | 8.54^ab^ | 10.1^a^ | 9.53^ab^ | 9.62^ab^ | 9.53^ab^ | 8.98^b^ | 8.13^c^ | 0.309 |  | 0.035 |
|  | *Enterococcus* | 8.63^a^ | 8.13^a^ | 6.85^b^ | 7.65^ab^ | 8.47^ab^ | 7.62^a^ | 7.96^ab^ | 0.379 |  | 0.007 |
|  | *Faecalibacterium* | 6.54^bc^ | 6.47^bc^ | 6.57^bc^ | 6.57^bc^ | 6.61^b^ | 5.69^c^ | 8.44^a^ | 0.315 |  | <0.001 |
|  | *Fusicatenibacter* | 6.08^b^ | 6.14^b^ | 5.84^b^ | ND | 6.12^b^ | 5.81^b^ | 7.71^a^ | 0.241 |  | 0.027 |
|  | *Lachnoanaerobaculum* | 7.66 | 8.19 | 7.69 | 7.57 | 8.05 | 7.60 | 7.78 | 0.256 |  | 0.523 |
|  | *Lachnospiraceae_unclassified* | 7.98^b^ | 8.63^a^ | 8.22^ab^ | 8.71^ab^ | 8.56^ab^ | 8.30^ab^ | 8.32^ab^ | 0.192 |  | 0.019 |
|  | *Lactobacillus* | 7.59 | 8.31 | 8.86 | 7.66 | 8.37 | 8.64 | 7.93 | 0.618 |  | 0.706 |
|  | *Lactococcus* | 7.37^ab^ | 6.66^ab^ | 6.84^ab^ | 6.19^ab^ | 7.85^a^ | 7.97^a^ | 5.79^d^ | 0.432 |  | 0.007 |
|  | *Leuconostoc* | 6.95 | 6.13 | 6.43 | 6.10 | 6.97 | 7.47 | ND | 0.453 |  | 0.093 |
|  | *Megamonas* | 7.62^ab^ | 7.77^abc^ | 7.95^ab^ | 6.79^bc^ | 7.63^abc^ | 5.96^c^ | 8.79^a^ | 0.371 |  | <0.001 |
|  | *Megasphaera* | 7.02^ab^ | 7.14^a^ | 8.19^ab^ | 7.32^ab^ | 7.67^a^ | 5.67^b^ | 6.82^a^ | 0.486 |  | 0.001 |
|  | *Mycoplasma* | 7.60 | 8.25 | 6.88 | 7.11 | 7.59 | 6.53 | 7.10 | 0.505 |  | 0.315 |
|  | *Parvimonas* | 7.19 | 7.89 | 7.40 | 7.59 | 7.81 | 7.57 | 6.97 | 0.289 |  | 0.331 |
|  | *Phascolarctobacterium* | 8.29 | 9.11 | 8.62 | 9.14 | 8.86 | 8.25 | 8.74 | 0.223 |  | 0.063 |
|  | *Romboutsia* | 8.61^abc^ | 9.81^a^ | 9.26^ab^ | 9.24^ab^ | 9.41^ab^ | 9.07^b^ | 7.24^c^ | 0.236 |  | 0.001 |
|  | *Sarcina* | 9.09^ab^ | 10.3^a^ | 8.37^ab^ | 8.18^ab^ | 9.91^a^ | 8.30^ab^ | 6.54^b^ | 0.554 |  | 0.003 |
|  | *Selenomonadaceae_unclassified* | 7.33^abc^ | 9.35^abc^ | 9.66^a^ | 7.37^c^ | 9.43^ab^ | 7.42^abc^ | 7.70^bc^ | 0.376 |  | 0.019 |
|  | *Sharpea* | 5.68 | ND | 6.58 | 6.06 | 6.42 | ND | ND | 0.415 |  | 0.132 |
|  | *Streptococcus* | 9.45^ab^ | 9.80^a^ | 9.31^ab^ | 9.22^ab^ | 9.49^ab^ | 9.23^ab^ | 8.75^b^ | 0.290 |  | 0.039 |
|  | *Terrisporobacter* | 8.60^ab^ | 9.62^a^ | 9.26^a^ | 8.91^a^ | 8.63^ab^ | 8.87^a^ | 7.16^b^ | 0.330 |  | <0.001 |
|  | *Turicibacter* | 9.13^ab^ | 9.52^a^ | 9.54^a^ | 9.67^a^ | 9.45^a^ | 8.68^a^ | 7.58^b^ | 0.324 |  | <0.001 |
|  | *Veillonella* | 8.65^ab^ | 9.19^ab^ | 9.28^a^ | 8.77^ab^ | 9.36^ab^ | 8.11^b^ | 8.78^ab^ | 0.297 |  | 0.030 |
|  | *Weissella* | 7.92 | 6.59 | 6.68 | 6.06 | 7.58 | 7.22 | 6.41 | 0.505 |  | 0.194 |
| Fusobacteria | | 9.43^b^ | 10.0^a^ | 9.37^b^ | 10.0^ab^ | 9.79^ab^ | 9.36^b^ | 9.16^b^ | 0.166 |  | 0.002 |
|  | *Fusobacterium* | 9.42^b^ | 10.0^a^ | 9.36^b^ | 10.0^ab^ | 9.76^ab^ | 9.35^b^ | 9.16^b^ | 0.164 |  | 0.002 |
|  | *Leptotrichia* | 6.73 | 7.35 | 7.13 | 6.77 | 7.92 | 6.28 | 6.19 | 0.453 |  | 0.085 |
| Proteobacteria | | 10.0^ab^ | 10.1^a^ | 9.59^ab^ | 10.3^ab^ | 10.2^ab^ | 10.0^a^ | 9.56^b^ | 0.184 |  | <0.001 |
|  | *Acinetobacter* | 7.28^ab^ | 7.32^ab^ | 6.24^b^ | 6.77^ab^ | 6.87^ab^ | 8.31^a^ | 6.73^ab^ | 0.293 |  | 0.009 |
|  | *Actinobacillus* | 8.87 | 8.80 | 8.49 | 9.15 | 8.24 | 8.89 | 8.59 | 0.307 |  | 0.535 |
|  | *Enterobacterales_unclassified* | 7.58^ab^ | 7.82^a^ | 7.32^ab^ | 7.80^ab^ | 7.84^ab^ | 7.91^ab^ | 7.12^b^ | 0.223 |  | 0.013 |
|  | *Enterobacteriaceae_unclassified* | 8.51 | 8.44 | 7.45 | 7.56 | 8.26 | 8.65 | 7.71 | 0.351 |  | 0.095 |
|  | *Erwiniaceae_unclassified* | 5.83 | ND | 5.90 | ND | 6.04 | 7.09 | ND | 0.367 |  | 0.372 |
|  | *Escherichia-Shigella* | 9.76 | 9.87 | 9.42 | 10.0 | 9.84 | 9.36 | 9.34 | 0.273 |  | 0.380 |
|  | *Kosakonia* | 6.99^ab^ | 6.46^ab^ | ND | 6.06^ab^ | 7.36^ab^ | 7.43^a^ | 5.36^b^ | 0.458 |  | 0.009 |
|  | *Pantoea* | 6.02 | 6.28 | 5.91 | 6.19 | 6.51 | 6.91 | 5.70 | 0.321 |  | 0.341 |
|  | *Pasteurellaceae_unclassified* | 8.08 | 8.44 | 8.03 | 8.14 | 8.04 | 7.82 | 8.17 | 0.321 |  | 0.917 |
|  | *Peptostreptococcaceae_unclassified* | 7.90 | 8.65 | 7.54 | 8.06 | 7.81 | 8.40 | 6.96 | 0.357 |  | 0.080 |
|  | *Pseudomonas* | 5.77^bc^ | 6.26^b^ | ND | 6.22^bc^ | 6.49^ab^ | 8.50^a^ | 5.37^c^ | 0.269 |  | 0.001 |
|  | *Rahnella1* | 6.04 | ND | ND | 6.10 | 6.28 | 7.47 | ND | 0.430 |  | 0.310 |
|  | *Sutterella* | 7.86^ab^ | 8.43^ab^ | 8.11^ab^ | 8.67^a^ | 8.31^ab^ | 7.19^b^ | 8.17^ab^ | 0.326 |  | 0.035 |
|  | *Yersiniaceae_unclassified* | 5.84 | ND | ND | ND | 6.10 | 6.87 | ND | 0.038 |  | 0.296 |
| Spirochaetes | | 7.04 | 6.98 | 6.51 | 7.08 | 6.84 | 6.90 | 6.65 | 0.519 |  | 0.986 |
|  | *Treponema* | 7.04 | 6.98 | 6.51 | 7.08 | 6.83 | 6.90 | 6.64 | 0.519 |  | 0.986 |
| Synergistetes | | 7.16 | 7.98 | 7.41 | 7.58 | 7.86 | 7.47 | 6.80 | 0.397 |  | 0.476 |
|  | *Fretibacterium* | 7.11 | 7.89 | 7.39 | 7.29 | 7.85 | 7.43 | 6.80 | 0.386 |  | 0.467 |
| ^1^ Values are means with pooled SEM, *n* = 4-6 animals per diet. Only taxa with >1% relative abundance in at least one sample were considered. The number of 16S rRNA gene copies per taxa was obtained by multiplying the total number of 16S rRNA gene copies with the relative abundance of the taxa with the assumption that each taxon has an equal number of 16S rRNA gene copies. Data were log_10_ transformed to achieve homogenous variance. A one-way ANOVA model was used to assess the effect of diet. Means in a row with a different letter differ (*P* ≤ 0.05). ND, not detected.  ^2^ *n* indicates the number of replicates. The different number of replicates resulted from either removing one pig that displayed coprophagy or the extracted DNA having low quality or concentration for 16S rRNA gene sequencing. | | | | | | | | | | | |

**Supplemental Table 6:** Predicted metabolic activity (according to PICRUSt) related to fermentation (i.e., carbohydrate and protein metabolism) in ileal digesta from growing pigs fed diets for seven days containing different test foods^1^

|  | | Diet | | | | | | | |  |  |  | |
| --- | --- | --- | --- | --- | --- | --- | --- | --- | --- | --- | --- | --- | --- |
| KEGG reference pathway | | Black bean | | Bread | Chickpea | Peanut | Pigeon pea | Sorghum | Wheat bran | SEM |  | *P* value | |
| Sample size, *n*^2^ | | 4 | | 5 | 4 | 5 | 6 | 6 | 6 |  |  |  | |
|  | | *log_10_ relative activity/g wet digesta* | | | | | | | |  |  |  | |
| Amino Acid Metabolism | | 6.96 | | 6.95 | 6.96 | 6.94 | 6.86 | 7.03 | 6.96 | 0.049 |  | 0.260 | |
|  | Alanine, aspartate, and glutamate metabolism | | 5.96 | 5.97 | 6.97 | 6.97 | 5.88 | 6.02 | 6.00 | 0.044 |  | 0.314 | |
|  | Amino acid related enzymes | | 6.14 | 6.15 | 6.16 | 6.14 | 6.07 | 6.19 | 6.18 | 0.044 |  | 0.437 | |
|  | Arginine and proline metabolism | | 6.03 | 6.03 | 6.00 | 5.99 | 5.92 | 6.13 | 6.00 | 0.055 |  | 0.191 | |
|  | Cysteine and methionine metabolism | | 6.00 | 5.99 | 6.00 | 5.98 | 5.91 | 6.05 | 6.00 | 0.045 |  | 0.362 | |
|  | Glycine, serine, and threonine metabolism | | 5.92 | 5.92 | 5.91 | 5.89 | 5.82 | 6.00 | 5.93 | 0.051 |  | 0.251 | |
|  | Histidine metabolism | | 5.71 | 5.72 | 5.75 | 5.74 | 5.61 | 5.76 | 5.70 | 0.046 |  | 0.206 | |
|  | Lysine biosynthesis | | 5.83 | 5.84 | 5.86 | 5.82 | 5.75 | 5.88 | 5.86 | 0.046 |  | 0.377 | |
|  | Lysine degradation | | 5.27^ab^ | 5.23^ab^ | 5.23^ab^ | 5.22^ab^ | 5.12^b^ | 5.44^a^ | 5.16^ab^ | 0.068 |  | 0.032 | |
|  | Phenylalanine metabolism | | 5.29 | 5.25 | 5.23 | 5.20 | 5.14 | 5.43 | 5.25 | 0.075 |  | 0.164 | |
|  | Phenylalanine, tyrosine, and tryptophan biosynthesis | | 5.84 | 5.85 | 5.86 | 5.84 | 5.75 | 5.89 | 5.90 | 0.051 |  | 0.383 | |
|  | Tryptophan metabolism | | 5.47^ab^ | 5.43^ab^ | 5.45^ab^ | 5.40^ab^ | 5.35^b^ | 5.63^a^ | 5.37^ab^ | 0.069 |  | 0.007 | |
|  | Tyrosine metabolism | | 5.60 | 5.57 | 5.59 | 5.55 | 5.49 | 5.69 | 5.53 | 0.052 |  | 0.134 | |
|  | Valine, leucine, and isoleucine biosynthesis | | 5.80 | 5.80 | 5.79 | 5.78 | 5.71 | 5.87 | 5.79 | 0.045 |  | 0.243 | |
|  | Valine, leucine, and isoleucine degradation | | 5.50^ab^ | 5.42^ab^ | 5.43^ab^ | 5.44^ab^ | 5.31^b^ | 5.62^a^ | 5.35 | 0.070 |  | 0.031 | |
| Carbohydrate Metabolism | | 7.03 | | 7.01 | 7.00 | 6.98 | 6.93 | 7.10 | 7.00 | 0.047 |  | 0.192 | |
|  | Amino sugar and nucleotide sugar metabolism | | 6.17 | 6.16 | 6.14 | 6.13 | 6.09 | 6.21 | 6.17 | 0.041 |  | 0.470 | |
|  | Ascorbate and aldarate metabolism | | 5.33^ab^ | 5.20^ab^ | 5.08^b^ | 5.20^ab^ | 5.10^b^ | 5.45^a^ | 5.15^ab^ | 0.093 |  | 0.040 | |
|  | Butanoate metabolism | | 5.88^ab^ | 5.84^ab^ | 5.84^ab^ | 5.86^ab^ | 5.72^b^ | 5.96^a^ | 5.78^ab^ | 0.052 |  | 0.027 | |
|  | C5-Branched dibasic acid metabolism | | 5.44 | 5.45 | 5.45 | 5.43 | 5.35 | 5.53 | 5.48 | 0.050 |  | 0.211 | |
|  | Citrate cycle (TCA cycle) | | 5.80 | 5.77 | 5.77 | 5.77 | 5.71 | 5.86 | 5.84 | 0.056 |  | 0.479 | |
|  | Fructose and mannose metabolism | | 6.00 | 5.95 | 5.95 | 5.92 | 5.91 | 6.06 | 5.99 | 0.051 |  | 0.351 | |
|  | Galactose metabolism | | 5.84 | 5.84 | 5.78 | 5.77 | 5.75 | 5.88 | 5.80 | 0.038 |  | 0.186 | |
|  | Glycolysis/Gluconeogenesis | | 6.06 | 6.03 | 6.04 | 6.00 | 5.96 | 6.11 | 6.02 | 0.044 |  | 0.247 | |
|  | Glyoxylate and dicarboxylate metabolism | | 5.72^ab^ | 5.67^ab^ | 5.65^ab^ | 5.69^ab^ | 5.56^b^ | 5.80^a^ | 5.67^ab^ | 0.057 |  | 0.050 | |
|  | Inositol phosphate metabolism | | 5.19^ab^ | 5.04^ab^ | 4.98^ab^ | 5.10^ab^ | 4.95^b^ | 5.29^a^ | 5.06^ab^ | 0.088 |  | 0.047 | |
|  | Pentose and glucuronate interconversions | | 5.78^ab^ | 5.71^ab^ | 5.67^ab^ | 5.72^ab^ | 5.59^b^ | 5.85^a^ | 5.66^ab^ | 0.052 |  | 0.009 | |
|  | Pentose phosphate pathway | | 5.91 | 5.89 | 5.89 | 5.86 | 5.81 | 5.98 | 5.88 | 0.047 |  | 0.211 | |
|  | Propanoate metabolism | | 5.76^ab^ | 5.70^ab^ | 5.71^ab^ | 5.72^ab^ | 5.60^b^ | 5.84^a^ | 5.67^ab^ | 0.056 |  | 0.048 | |
|  | Pyruvate metabolism | | 6.03 | 6.00 | 5.99 | 5.97 | 5.92 | 6.10 | 5.96 | 0.049 |  | 0.177 | |
|  | Starch and sucrose metabolism | | 5.97 | 5.95 | 5.96 | 5.92 | 5.87 | 6.03 | 5.96 | 0.042 |  | 0.175 | |
| Metabolism of Other Amino Acids | | 6.21 | | 6.19 | 6.20 | 6.17 | 6.10 | 6.29 | 6.19 | 0.051 |  | 0.198 | |
|  | beta-Alanine metabolism | | 5.30^ab^ | 5.28^ab^ | 5.26^ab^ | 5.27^ab^ | 5.19^b^ | 5.47^a^ | 5.36^ab^ | 0.067 |  | 0.049 | |
|  | Cyanoamino acid metabolism | | 5.44 | 5.40 | 5.40 | 5.39 | 5.30 | 5.50 | 5.39 | 0.053 |  | 0.170 | |
|  | D-Alanine metabolism | | 5.09 | 5.08 | 5.09 | 5.06 | 5.00 | 5.12 | 5.07 | 0.039 |  | 0.416 | |
|  | D-Arginine and D-ornithine metabolism | | 3.72 | 3.80 | 3.91 | 3.70 | 3.71 | 3.83 | 3.93 | 0.092 |  | 0.403 | |
|  | D-Glutamine and D-glutamate metabolism | | 5.16 | 5.18 | 5.16 | 5.16 | 5.08 | 5.20 | 5.17 | 0.047 |  | 0.565 | |
|  | Glutathione metabolism | | 5.53^ab^ | 5.43^ab^ | 5.46^ab^ | 5.49^ab^ | 5.33^b^ | 5.60^a^ | 5.41^ab^ | 0.056 |  | 0.015 | |
|  | Phosphonate and phosphinate metabolism | | 4.82 | 4.79 | 4.73 | 4.77 | 4.66 | 4.92 | 4.65 | 0.016 |  | 0.058 | |
|  | Selenocompound metabolism | | 5.57 | 5.56 | 5.57 | 5.53 | 5.48 | 5.62 | 5.57 | 0.049 |  | 0.454 | |
|  | Taurine and hypotaurine metabolism | | 5.09 | 5.07 | 5.11 | 5.06 | 4.98 | 5.13 | 5.02 | 0.051 |  | 0.271 | |
| Digestive System | | 4.59^ab^ | | 4.68^ab^ | 4.65^ab^ | 4.60^ab^ | 4.62^b^ | 4.63^ab^ | 4.86^a^ | 0.075 |  | 0.008 | |
|  | Carbohydrate digestion and absorption | | 4.28 | 4.31 | 4.35 | 4.17 | 4.27 | 4.35 | 4.40 | 0.080 |  | 0.571 | |
|  | Protein digestion and absorption | | 4.01 | 4.24 | 4.19 | 4.25 | 4.14 | 4.00 | 4.49 | 0.107 |  | 0.100 | |
| Metabolism | | 6.46^ab^ | | 6.40^ab^ | 6.36^ab^ | 6.42^ab^ | 6.28^b^ | 6.51^a^ | 6.37^ab^ | 0.053 |  | 0.044 | |
|  | Amino acid metabolism | | 5.45 | 5.41 | 5.36 | 5.39 | 5.32 | 5.50 | 5.36 | 0.056 |  | 0.213 | |
|  | Carbohydrate metabolism | | 5.14 | 5.12 | 5.09 | 5.04 | 5.01 | 5.24 | 5.08 | 0.060 |  | 0.115 | |
| ^1^ Values are means with pooled SEM, *n* = 4-6 per diet. Data were log_10_ transformed to achieve homogenous variance. A one-way ANOVA model was used to assess the diet effect. Means in a row with a different letter differ (*P* ≤ 0.05). PICRUSt, Phylogenetic Investigation of Communities by Reconstruction of Unobserved States.  ^2^ *n* indicates the number of replicates. The different numbers of replicates resulted from either removing one pig that displayed coprophagy or the extracted DNA having low quality or concentration for 16S rRNA gene sequencing. | | | | | | | | | | | | |  |

**Supplementary Table 7:** Pearson’s correlation coefficients between fermentation outcomes (OM fermentability and organic acid production) and the number of bacteria in the ileal inoculum per fiber substrate after *in vitro* fermentation^1^

| Fiber  substrate | Bacteria, log_10_ 16S rRNA gene copies/g wet digesta | | OM fermentability, % | Organic acid production, mmol/kg DM substrate | | | | |
| --- | --- | --- | --- | --- | --- | --- | --- | --- |
|  |  |  |  | Total | Formic acid | Acetic acid | Propionic acid | Lactic acid |
| AG | Total bacteria | | 0.46 (0.300) | 0.01 (0.987) | -0.63 (0.128) | 0.34 (0.461) | 0.21 (0.659) | 0.41 (0.357) |
|  | Actinobacteriota | | 0.39 (0.392) | 0.22 (0.635) | -0.77 (0.044) | 0.56 (0.192) | -0.24 (0.603) | 0.58 (0.175) |
|  |  | *Actinomyces* | 0.20 (0.660) | 0.07 (0.887) | -0.66 (0.104) | 0.32 (0.489) | 0.12 (0.799) | 0.51 (0.237) |
|  |  | *Bifidobacterium* | -0.02 (0.959) | 0.29 (0.529) | -0.64 (0.121) | 0.82 (0.025) | -0.56 (0.193) | 0.29 (0.521) |
|  |  | *Collinsella* | 0.52 (0.230) | 0.48 (0.280) | 0.21 (0.657) | 0.50 (0.250) | 0.53 (0.224) | -0.06 (0.895) |
|  | Bacteroidota | | 0.65 (0.155) | 0.37 (0.420) | -0.20 (0.675) | 0.54 (0.210) | 0.47 (0.292) | 0.25 (0.594) |
|  |  | *Alloprevotella* | 0.28 (0.542) | -0.34 (0.450) | 0.47 (0.287) | -0.06 (0.893) | 0.78 (0.040) | -0.65 (0.133) |
|  |  | *Bacteroides* | 0.72 (0.070) | -0.15 (0.753) | -0.56 (0.195) | 0.27 (0.558) | 0.34 (0.458) | 0.17 (0.710) |
|  |  | *Muribaculaceae_unclassified* | 0.81 (0.029) | -0.08 (0.867) | 0.01 (0.978) | 0.54 (0.207) | 0.55 (0.204) | -0.45 (0.309) |
|  |  | *Parabacteroides* | 0.47 (0.284) | -0.41 (0.360) | -0.52 (0.237) | -0.18 (0.706) | 0.35 (0.447) | 0.17 (0.720) |
|  |  | *Porphyromonas* | 0.26 (0.567) | -0.22 (0.632) | -0.47 (0.286) | 0.10 (0.832) | 0.30 (0.509) | 0.25 (0.594) |
|  |  | *Prevotella* | 0.06 (0.901) | 0.55 (0.199) | 0.02 (0.964) | 0.59 (0.160) | 0.34 (0.457) | 0.24 (0.603) |
|  |  | *Prevotellaceae_NK3B31_group* | 0.32 (0.485) | 0.60 (0.159) | -0.10 (0.837) | 0.48 (0.274) | 0.31 (0.496) | 0.38 (0.407) |
|  |  | *Prevotellaceae_UCG003* | 0.46 (0.296) | -0.33 (0.467) | 0.63 (0.132) | -0.31 (0.495) | 0.71 (0.076) | -0.57 (0.179) |
|  |  | *Prevotellaceae_unclassified* | 0.67 (0.099) | 0.02 (0.964) | -0.40 (0.368) | 0.42 (0.349) | 0.51 (0.243) | 0.10 (0.834) |
|  | Firmicutes | | 0.34 (0.458) | -0.04 (0.981) | -0.76 (0.049) | 0.32 (0.491) | 0.08 (0.867) | 0.47 (0.283) |
|  |  | *Agathobacter* | 0.11 (0.813) | 0.48 (0.275) | 0.33 (0.474) | 0.23 (0.619) | 0.26 (0.570) | -0.09 (0.851) |
|  |  | *Anaerovibrio* | 0.11 (0.807) | 0.83 (0.020) | 0.41 (0.365) | 0.53 (0.221) | 0.08 (0.864) | 0.18 (0.697) |
|  |  | *Blautia* | 0.02 (0.961) | 0.64 (0.123) | 0.81 (0.028) | 0.23 (0.621) | 0.31 (0.504) | -0.13 (0.775) |
|  |  | *Clostridium_sensu_stricto_1* | 0.47 (0.291) | 0.09 (0.854) | -0.59 (0.167) | 0.15 (0.749) | 0.12 (0.795) | 0.56 (0.195) |
|  |  | *Enterococcus* | 0.12 (0.797) | -0.41 (0.360) | 0.11 (0.811) | 0.11 (0.818) | 0.64 (0.124) | -0.65 (0.113) |
|  |  | *Faecalibacterium* | -0.31 (0.505) | 0.68 (0.093) | 0.55 (0.202) | 0.37 (0.418) | -0.01 (0.975) | -0.01 (0.987) |
|  |  | *Lachnoanaerobaculum* | -0.13 (0.783) | 0.20 (0.672) | -0.26 (0.578) | 0.17 (0.713) | 0.31 (0.501) | 0.35 (0.436) |
|  |  | *Lachnospiraceae_unclassified* | 0.60 (0.152) | 0.42 (0.351) | 0.15 (0.748) | 0.22 (0.635) | 0.53 (0.224) | 0.32 (0.478) |
|  |  | *Lactobacillus* | -0.54 (0.215) | 0.15 (0.743) | -0.41 (0.362) | -0.21 (0.644) | -0.59 (0.159) | 0.79 (0.033) |
|  |  | *Lactococcus* | -0.19 (0.677) | -0.67 (0.100) | -0.45 (0.311) | -0.22 (0.634) | -0.13 (0.788) | -0.08 (0.858) |
|  |  | *Megamonas* | -0.45 (0.315) | 0.61 (0.145) | 0.02 (0.965) | 0.50 (0.258) | -0.22 (0.632) | 0.23 (0.612) |
|  |  | *Megasphaera* | 0.05 (0.920) | 0.54 (0.209) | -0.61 (0.146) | 0.85 (0.017) | -0.44 (0.322) | 0.56 (0.194) |
|  |  | *Parvimonas* | 0.42 (0.349) | -0.13 (0.779) | -0.44 (0.320) | 0.00 (0.999) | 0.33 (0.470) | 0.36 (0.429) |
|  |  | *Peptostreptococcaceae_unclassified* | 0.48 (0.263) | -0.62 (0.137) | -0.32 (0.482) | -0.50 (0.257) | 0.49 (0.262) | -0.10 (0.835) |
|  |  | *Phascolarctobacterium* | 0.55 (0.196) | 0.60 (0.151) | 0.04 (0.930) | 0.46 (0.294) | 0.42 (0.350) | 0.36 (0.425) |
|  |  | *Romboutsia* | 0.43 (0.338) | -0.26 (0.566) | -0.71 (0.072) | 0.01 (0.982) | 0.08 (0.857) | 0.37 (0.418) |
|  |  | *Sarcina* | 0.24 (0.597) | -0.40 (0.368) | -0.71 (0.072) | 0.08 (0.859) | 0.26 (0.575) | 0.12 (0.804) |
|  |  | *Selenomonadaceae_unclassified* | -0.30 (0.511) | 0.41 (0.355) | -0.67 (0.101) | 0.38 (0.394) | -0.39 (0.388) | 0.85 (0.016) |
|  |  | *Streptococcus* | 0.28 (0.545) | -0.43 (0.331) | -0.75 (0.052) | -0.02 (0.970) | 0.23 (0.620) | 0.13 (0.782) |
|  |  | *Terrisporobacter* | 0.37 (0.416) | -0.33 (0.470) | -0.75 (0.051) | -0.15 (0.755) | -0.02 (0.973) | 0.35 (0.445) |
|  |  | *Turicibacter* | 0.56 (0.193) | -0.18 (0.697) | -0.79 (0.036) | 0.32 (0.491) | -0.01 (0.977) | 0.28 (0.549) |
|  |  | *Veillonella* | -0.06 (0.895) | 0.57 (0.179) | -0.56 (0.194) | 0.74 (0.055) | -0.24 (0.609) | 0.65 (0.115) |
|  | Fusobacteriota | | 0.80 (0.032) | -0.01 (0.977) | -0.28 (0.540) | 0.26 (0.581) | 0.58 (0.172) | 0.09 (0.845) |
|  |  | *Fusobacterium* | 0.81 (0.029) | -0.01 (0.984) | -0.27 (0.561) | 0.24 (0.601) | 0.59 (0.164) | 0.09 (0.853) |
|  |  | *Leptotrichia* | 0.12 (0.804) | 0.14 (0.769) | -0.71 (0.075) | 0.59 (0.164) | -0.22 (0.938) | 0.23 (0.288) |
|  | Proteobacteria | | 0.80 (0.031) | -0.41 (0.359) | -0.12 (0.796) | 0.08 (0.870) | 0.69 (0.089) | -0.32 (0.477) |
|  |  | *Acinetobacter* | 0.03 (0.955) | -0.77 (0.043) | 0.24 (0.612) | -0.84 (0.018) | 0.45 (0.308) | -0.48 (0.273) |
|  |  | *Actinobacillus* | 0.69 (0.086) | -0.42 (0.344) | 0.30 (0.515) | -0.48 (0.280) | 0.50 (0.253) | -0.55 (0.198) |
|  |  | *Enterobacterales_unclassified* | 0.56 (0.187) | -0.56 (0.190) | -0.22 (0.642) | -0.25 (0.593) | 0.53 (0.217) | -0.17 (0.723) |
|  |  | *Enterobacteriaceae_unclassified* | -0.09 (0.855) | -0.82 (0.024) | -0.08 (0.860) | -0.57 (0.180) | 0.44 (0.319) | -0.47 (0.290) |
|  |  | *Escherichia_Shigella* | 0.82 (0.023) | -0.14 (0.765) | -0.28 (0.539) | 0.42 (0.346) | 0.60 (0.158) | -0.21 (0.655) |
|  |  | *Pantoea* | 0.17 (0.715) | -0.54 (0.208) | -0.08 (0.870) | -0.46 (0.297) | 0.28 (0.546) | -0.06 (0.903) |
|  |  | *Pasteurellaceae_unclassified* | 0.28 (0.548) | 0.31 (0.502) | -0.04 (0.934) | 0.18 (0.699) | 0.47 (0.291) | -0.10 (0.826) |
|  |  | *Sutterella* | 0.52 (0.235) | 0.61 (0.146) | -0.09 (0.855) | 0.72 (0.068) | 0.26 (0.579) | 0.25 (0.582) |
|  | Spirochaetota | | 0.78 (0.039) | -0.63 (0.129) | 0.08 (0.866) | -0.20 (0.675) | 0.82 (0.023) | -0.69 (0.084) |
|  |  | *Treponema* | 0.78 (0.038) | -0.64 (0.124) | 0.07 (0.883) | -0.20 (0.660) | 0.82 (0.026) | -0.69 (0.087) |
|  | Synergistota | | 0.42 (0.344) | -0.12 (0.792) | -0.53 (0.216) | 0.07 (0.876) | 0.29 (0.525) | 0.38 (0.406) |
|  |  | *Fretibacterium* | 0.19 (0.683) | -0.13 (0.785) | -0.59 (0.164) | 0.05 (0.917) | 0.18 (0.703) | 0.45 (0.316) |
|  | Mycoplasmatota | |  |  |  |  |  |  |
|  |  | *Mycoplasma* | 0.22 (0.633) | -0.07 (0.889) | -0.32 (0.487) | 0.24 (0.610) | 0.51 (0.245) | -0.07 (0.888) |
|  |  | |  |  |  |  |  |  |
| Cellulose | Total bacteria | | 0.03 (0.941) | 0.17 (0.717) | 0.66 (0.107) | -0.21 (0.656) | 0.39 (0.383) | 0.31 (0.500) |
|  | Actinobacteriota | | 0.35 (0.439) | 0.00 (0.993) | 0.31 (0.503) | -0.39 (0.381) | 0.79 (0.034) | 0.39 (0.393) |
|  |  | *Actinomyces* | -0.15 (0.744) | 0.06 (0.897) | 0.79 (0.036) | -0.26 (0.580) | 0.39 (0.386) | 0.13 (0.780) |
|  |  | *Bifidobacterium* | 0.14 (0.758) | -0.53 (0.219) | -0.07 (0.883) | -0.70 (0.077) | 0.67 (0.099) | 0.34 (0.458) |
|  |  | *Collinsella* | 0.31 (0.496) | -0.56 (0.194) | 0.50 (0.257) | -0.72 (0.068) | -0.05 (0.915) | 0.04 (0.938) |
|  | Bacteroidota | | 0.24 (0.603) | -0.16 (0.727) | 0.70 (0.079) | -0.52 (0.231) | 0.22 (0.633) | 0.29 (0.531) |
|  |  | *Alloprevotella* | -0.54 (0.210) | 0.20 (0.667) | 0.22 (0.632) | 0.25 (0.596) | -0.85 (0.016) | 0.30 (0.508) |
|  |  | *Bacteroides* | 0.26 (0.575) | 0.19 (0.687) | 0.46 (0.297) | -0.19 (0.682) | 0.30 (0.515) | 0.39 (0.392) |
|  |  | *Muribaculaceae_unclassified* | 0.18 (0.706) | -0.15 (0.756) | 0.09 (0.851) | -0.37 (0.407) | -0.19 (0.689) | 0.65 (0.115) |
|  |  | *Parabacteroides* | 0.03 (0.956) | 0.48 (0.276) | 0.47 (0.291) | 0.21 (0.648) | 0.12 (0.804) | 0.15 (0.749) |
|  |  | *Porphyromonas* | -0.36 (0.426) | 0.42 (0.348) | 0.64 (0.120) | 0.15 (0.747) | 0.06 (0.892) | 0.31 (0.492) |
|  |  | *Prevotella* | -0.28 (0.541) | -0.46 (0.293) | 0.82 (0.023) | -0.62 (0.139) | -0.01 (0.989) | 0.03 (0.954) |
|  |  | *Prevotellaceae_NK3B31_group* | 0.25 (0.585) | -0.49 (0.263) | 0.76 (0.048) | -0.72 (0.068) | 0.27 (0.565) | -0.13 (0.788) |
|  |  | *Prevotellaceae_UCG003* | -0.17 (0.722) | 0.60 (0.150) | -0.09 (0.853) | -0.61 (0.144) | -0.73 (0.065) | 0.39 (0.384) |
|  |  | *Prevotellaceae_unclassified* | 0.14 (0.769) | -0.12 (0.794) | 0.68 (0.090) | -0.46 (0.296) | 0.14 (0.763) | 0.27 (0.557) |
|  | Firmicutes | | 0.04 (0.929) | 0.06 (0.906) | 0.65 (0.112) | -0.29 (0.528) | 0.48 (0.272) | 0.17 (0.711) |
|  |  | *Agathobacter* | 0.35 (0.443) | -0.79 (0.035) | 0.25 (0.586) | -0.73 (0.063) | -0.08 (0.869) | -0.42 (0.343) |
|  |  | *Anaerovibrio* | 0.33 (0.475) | -0.56 (0.194) | 0.21 (0.650) | -0.62 (0.139) | 0.14 (0.760) | -0.03 (0.950) |
|  |  | *Blautia* | 0.06 (0.904) | -0.32 (0.479) | 0.09 (0.844) | -0.24 (0.605) | -0.32 (0.478) | -0.04 (0.926) |
|  |  | *Clostridium_sensu_stricto_1* | 0.30 (0.515) | 0.25 (0.587) | 0.58 (0.174) | -0.12 (0.800) | 0.53 (0.221) | 0.14 (0.765) |
|  |  | *Enterococcus* | -0.60 (0.154) | -0.29 (0.523) | 0.35 (0.445) | -0.21 (0.649) | -0.72 (0.067) | 0.07 (0.881) |
|  |  | *Faecalibacterium* | -0.08 (0.873) | -0.63 (0.132) | 0.09 (0.852) | -0.48 (0.274) | -0.14 (0.764) | -0.20 (0.663) |
|  |  | *Lachnoanaerobaculum* | -0.45 (0.312) | -0.24 (0.600) | 0.97 (<0.001) | -0.35 (0.440) | -0.01 (0.982) | -0.28 (0.543) |
|  |  | *Lachnospiraceae_unclassified* | 0.21 (0.646) | 0.32 (0.489) | 0.62 (0.138) | -0.01 (0.983) | 0.09 (0.847) | 0.31 (0.501) |
|  |  | *Lactobacillus* | -0.18 (0.703) | 0.45 (0.313) | 0.15 (0.746) | 0.40 (0.377) | 0.55 (0.201) | -0.18 (0.697) |
|  |  | *Lactococcus* | -0.58 (0.168) | 0.53 (0.224) | -0.06 (0.895) | 0.53 (0.223) | -0.11 (0.822) | 0.26 (0.570) |
|  |  | *Megamonas* | -0.15 (0.752) | -0.85 (0.015) | 0.32 (0.478) | -0.77 (0.044) | 0.16 (0.730) | -0.36 (0.434) |
|  |  | *Megasphaera* | 0.22 (0.641) | -0.50 (0.252) | 0.26 (0.575) | -0.76 (0.045) | 0.78 (0.040) | 0.25 (0.586) |
|  |  | *Parvimonas* | -0.05 (0.913) | 0.47 (0.282) | 0.64 (0.123) | 0.16 (0.738) | 0.19 (0.677) | 0.22 (0.629) |
|  |  | *Peptostreptococcaceae_unclassified* | 0.12 (0.801) | 0.46 (0.299) | 0.31 (0.499) | 0.32 (0.481) | -0.13 (0.776) | -0.08 (0.861) |
|  |  | *Phascolarctobacterium* | 0.35 (0.442) | -0.15 (0.744) | 0.67 (0.101) | -0.47 (0.290) | 0.24 (0.598) | 0.16 (0.736) |
|  |  | *Romboutsia* | 0.16 (0.732) | 0.37 (0.407) | 0.40 (0.370) | 0.06 (0.906) | 0.42 (0.348) | 0.20 (0.667) |
|  |  | *Sarcina* | -0.25 (0.596) | 0.04 (0.935) | 0.62 (0.138) | -0.17 (0.712) | 0.10 (0.826) | 0.05 (0.911) |
|  |  | *Selenomonadaceae_unclassified* | -0.18 (0.704) | -0.14 (0.769) | 0.65 (0.112) | -0.35 (0.443) | 0.68 (0.093) | -0.10 (0.825) |
|  |  | *Streptococcus* | -0.07 (0.882) | -0.01 (0.988) | 0.56 (0.187) | -0.21 (0.659) | 0.17 (0.718) | -0.08 (0.863) |
|  |  | *Terrisporobacter* | 0.34 (0.461) | 0.26 (0.568) | 0.27 (0.559) | 0.01 (0.981) | 0.48 (0.280) | -0.03 (0.954) |
|  |  | *Turicibacter* | 0.33 (0.463) | 0.12 (0.794) | 0.24 (0.611) | -0.24 (0.205) | 0.55 (0.205) | 0.38 (0.406) |
|  |  | *Veillonella* | -0.05 (0.907) | -0.46 (0.297) | 0.64 (0.118) | -0.72 (0.070) | 0.62 (0.137) | 0.08 (0.858) |
|  | Fusobacteriota | | 0.27 (0.564) | 0.12 (0.792) | 0.60 (0.158) | -0.26 (0.610) | 0.11 (0.814) | 0.32 (0.486) |
|  |  | *Fusobacterium* | 0.29 (0.531) | 0.12 (0.805) | 0.59 (0.161) | -0.24 (0.603) | 0.11 (0.820) | 0.30 (0.512) |
|  |  | *Leptotrichia* | -0.27 (0.559) | -0.08 (0.863) | 0.67 (0.099) | -0.39 (0.383) | 0.44 (0.327) | 0.32 (0.480) |
|  | Proteobacteria | | 0.01 (0.978) | 0.38 (0.38) | 0.29 (0.535) | 0.12 (0.792) | -0.26 (0.568) | 0.54 (0.214) |
|  |  | *Acinetobacter* | -0.31 (0.505) | 0.58 (0.170) | -0.09 (0.854) | 0.76 (0.049) | -0.67 (0.100) | -0.20 (0.665) |
|  |  | *Actinobacillus* | 0.67 (0.099) | 0.17 (0.723) | -0.37 (0.419) | 0.18 (0.697) | -0.32 (0.491) | -0.07 (0.886) |
|  |  | *Enterobacterales_unclassified* | -0.10 (0.832) | 0.65 (0.117) | 0.27 (0.561) | 0.44 (0.321) | -0.22 (0.637) | 0.35 (0.435) |
|  |  | *Enterobacteriaceae_unclassified* | -0.59 (0.166) | 0.26 (0.575) | 0.20 (0.667) | 0.41 (0.362) | -0.64 (0.123) | -0.22 (0.630) |
|  |  | *Escherichia_Shigella* | 0.20 (0.671) | -0.12 (0.794) | 0.43 (0.35) | -0.43 (0.340) | -0.03 (0.951) | 0.43 (0.342) |
|  |  | *Pantoea* | -0.32 (0.483) | 0.87 (0.010) | 0.09 (0.849) | 0.79 (0.034) | -0.25 (0.588) | 0.27 (0.562) |
|  |  | *Pasteurellaceae_unclassified* | 0.24 (0.610) | -0.62 (0.138) | 0.72 (0.070) | -0.71 (0.072) | 0.01 (0.991) | -0.44 (0.327) |
|  |  | *Sutterella* | 0.37 (0.412) | -0.46 (0.303) | 0.51 (0.241) | -0.75 (0.052) | 0.33 (0.476) | 0.24 (0.608) |
|  | Spirochaetota | | 0.12 (0.806) | 0.15 (0.742) | 0.09 (0.851) | 0.07 (0.879) | -0.56 (0.195) | 0.22 (0.631) |
|  |  | *Treponema* | 0.13 (0.783) | 0.16 (0.738) | 0.08 (0.864) | 0.07 (0.876) | -0.55 (0.205) | 0.22 (0.642) |
|  | Synergistetes | | -0.03 (0.957) | 0.37 (0.412) | 0.66 (0.108) | 0.04 (0.931) | 0.26 (0.578) | 0.21 (0.648) |
|  |  | *Fretibacterium* | -0.22 (0.634) | 0.34 (0.454) | 0.71 (0.076) | 0.06 (0.892) | 0.26 (0.579) | 0.12 (0.803) |
|  | Mycoplasmatota | |  |  |  |  |  |  |
|  |  | *Mycoplasma* | -0.17 (0.709) | -0.55 (0.203) | 0.79 (0.035) | -0.65 (0.116) | -0.14 (0.769) | -0.25 (0.585) |
|  |  |  |  |  |  |  |  |  |
| FOS | Total bacteria | | 0.36 (0.431) | 0.77 (0.043) | 0.05 (0.911) | 0.64 (0.119) | 0.20 (0.661) | 0.16 (0.724) |
|  | Actinobacteriota | | 0.33 (0.472) | 0.93 (0.002) | 0.28 (0.540) | 0.71 (0.074) | -0.23 (0.613) | 0.05 (0.915) |
|  |  | *Actinomyces* | 0.22 (0.633) | 0.63 (0.126) | -0.10 (0.827) | 0.75 (0.054) | 0.14 (0.770) | 0.39 (0.387) |
|  |  | *Bifidobacterium* | 0.09 (0.851) | 0.58 (0.171) | -0.16 (0.738) | 0.44 (0.319) | -0.39 (0.384) | 0.35 (0.436) |
|  |  | *Collinsella* | 0.71 (0.074) | 0.49 (0.270) | -0.29 (0.525) | -0.29 (0.522) | 0.47 (0.291) | 0.39 (0.385) |
|  | Bacteroidota | | 0.63 (0.126) | 0.81 (0.028) | -0.19 (0.844) | 0.17 (0.715) | 0.40 (0.374) | 0.28 (0.542) |
|  |  | *Alloprevotella* | 0.01 (0.991) | -0.28 (0.536) | -0.56 (0.192) | -0.53 (0.219) | 0.84 (0.017) | 0.09 (0.849) |
|  |  | *Bacteroides* | 0.57 (0.182) | 0.81 (0.028) | 0.18 (0.707) | 0.45 (0.310) | 0.34 (0.462) | -0.07 (0.886) |
|  |  | *Muribaculaceae_unclassified* | 0.58 (0.176) | 0.54 (0.209) | -0.25 (0.592) | -0.31 (0.491) | 0.63 (0.130) | -0.02 (0.973) |
|  |  | *Parabacteroides* | 0.31 (0.493) | 0.45 (0.309) | 0.24 (0.606) | 0.53 (0.220) | 0.32 (0.479) | -0.17 (0.721) |
|  |  | *Porphyromonas* | 0.04 (0.940) | 0.41 (0.365) | -0.11 (0.817) | 0.55 (0.200) | 0.33 (0.466) | 0.15 (0.753) |
|  |  | *Prevotella* | 0.22 (0.628) | 0.34 (0.449) | -0.61 (0.144) | 0.07 (0.885) | 0.34 (0.457) | 0.79 (0.036) |
|  |  | *Prevotellaceae_NK3B31_group* | 0.60 (0.157) | 0.61 (0.147) | -0.19 (0.691) | 0.15 (0.743) | 0.22 (0.638) | 0.54 (0.215) |
|  |  | *Prevotellaceae_UCG003* | -0.01 (0.987) | -0.24 (0.609) | 0.00 (0.995) | -0.64 (0.123) | 0.64 (0.123) | -0.47 (0.288) |
|  |  | *Prevotellaceae_unclassified* | 0.76 (0.100) | 0.76 (0.048) | -0.15 (0.741) | 0.30 (0.511) | 0.52 (0.229) | 0.27 (0.561) |
|  | Firmicutes | | 0.35 (0.438) | 0.74 (0.056) | 0.04 (0.928) | 0.76 (0.045) | 0.10 (0.829) | 0.25 (0.595) |
|  |  | *Agathobacter* | 0.57 (0.186) | 0.08 (0.865) | -0.27 (0.560) | -0.38 (0.402) | 0.20 (0.665) | 0.44 (0.328) |
|  |  | *Anaerovibrio* | 0.30 (0.515) | 0.28 (0.542) | -0.15 (0.756) | -0.37 (0.418) | -0.04 (0.938) | 0.34 (0.460) |
|  |  | *Blautia* | 0.07 (0.881) | -0.15 (0.746) | 0.26 (0.572) | -0.72 (0.067) | 0.18 (0.701) | 0.21 (0.656) |
|  |  | *Clostridium_sensu_stricto_1* | 0.42 (0.354) | 0.78 (0.039) | 0.37 (0.419) | 0.66 (0.106) | 0.03 (0.954) | -0.03 (0.945) |
|  |  | *Enterococcus* | 0.17 (0.722) | -0.24 (0.611) | -0.86 (0.014) | -0.28 (0.539) | 0.83 (0.021) | 0.50 (0.250) |
|  |  | *Faecalibacterium* | -0.06 (0.904) | -0.21 (0.658) | -0.45 (0.314) | -0.48 (0.273) | -0.05 (0.917) | 0.51 (0.247) |
|  |  | *Lachnoanaerobaculum* | 0.13 (0.787) | 0.17 (0.708) | -0.52 (0.236) | 0.44 (0.327) | 0.32 (0.488) | 0.77 (0.043) |
|  |  | *Lachnospiraceae_unclassified* | 0.37 (0.418) | 0.60 (0.153) | 0.19 (0.675) | 0.00 (0.994) | 0.32 (0.478) | -0.06 (0.901) |
|  |  | *Lactobacillus* | -0.57 (0.183) | 0.00 (0.999) | 0.39 (0.381) | 0.73 (0.061) | -0.68 (0.093) | -0.04 (0.934) |
|  |  | *Lactococcus* | -0.47 (0.284) | -0.19 (0.686) | -0.10 (0.835) | 0.44 (0.328) | 0.04 (0.935) | -0.11 (0.816) |
|  |  | *Megamonas* | 0.02 (0.972) | -0.02 (0.974) | -0.56 (0.190) | 0.02 (0.962) | -0.17 (0.719) | 0.82 (0.024) |
|  |  | *Megasphaera* | 0.20 (0.670) | 0.76 (0.049) | -0.08 (0.856) | 0.54 (0.212) | -0.37 (0.409) | 0.45 (0.312) |
|  |  | *Parvimonas* | 0.22 (0.631) | 0.54 (0.210) | 0.18 (0.706) | 0.55 (0.201) | 0.27 (0.563) | -0.03 (0.949) |
|  |  | *Peptostreptococcaceae_unclassified* | 0.42 (0.350) | 0.19 (0.682) | 0.29 (0.527) | 0.30 (0.515) | 0.45 (0.307) | -0.31 (0.497) |
|  |  | *Phascolarctobacterium* | 0.57 (0.180) | 0.72 (0.069) | 0.04 (0.938) | 0.03 (0.941) | 0.26 (0.575) | 0.23 (0.628) |
|  |  | *Romboutsia* | 0.31 (0.501) | 0.64 (0.124) | 0.34 (0.459) | 0.71 (0.073) | 0.07 (0.876) | -0.14 (0.762) |
|  |  | *Sarcina* | 0.30 (0.513) | 0.41 (0.365) | -0.22 (0.628) | 0.65 (0.111) | 0.38 (0.406) | 0.32 (0.486) |
|  |  | *Selenomonadaceae_unclassified* | -0.12 (0.805) | 0.47 (0.286) | -0.07 (0.885) | 0.87 (0.011) | -0.39 (0.388) | 0.55 (0.196) |
|  |  | *Streptococcus* | 0.42 (0.348) | 0.42 (0.344) | -0.09 (0.848) | 0.68 (0.096) | 0.32 (0.478) | 0.24 (0.601) |
|  |  | *Terrisporobacter* | 0.40 (0.377) | 0.56 (0.194) | 0.46 (0.296) | 0.72 (0.067) | -0.03 (0.941) | -0.21 (0.657) |
|  |  | *Turicibacter* | 0.46 (0.300) | 0.83 (0.022) | 0.29 (0.533) | 0.64 (0.123) | 0.03 (0.943) | -0.11 (0.809) |
|  |  | *Veillonella* | 0.15 (0.753) | 0.66 (0.109) | -0.28 (0.543) | 0.62 (0.141) | -0.20 (0.673) | 0.69 (0.085) |
|  | Fusobacteriota | | 0.68 (0.096) | 0.75 (0.050) | 0.08 (0.862) | 0.22 (0.643) | 0.52 (0.229) | 0.00 (0.999) |
|  |  | *Fusobacterium* | 0.69 (0.084) | 0.75 (0.051) | 0.09 (0.841) | 0.20 (0.666) | 0.53 (0.226) | -0.01 (0.986) |
|  |  | *Leptotrichia* | 0.09 (0.843) | 0.66 (0.110) | -0.29 (0.536) | 0.72 (0.067) | 0.06 (0.898) | 0.51 (0.240) |
|  | Proteobacteria | | 0.45 (0.311) | 0.44 (0.321) | -0.02 (0.968) | -0.03 (0.943) | 0.71 (0.074) | -0.23 (0.622) |
|  |  | *Acinetobacter* | -0.13 (0.779) | -0.59 (0.167) | 0.07 (0.887) | -0.20 (0.674) | 0.44 (0.317) | -0.39 (0.386) |
|  |  | *Actinobacillus* | 0.64 (0.118) | 0.02 (0.970) | 0.48 (0.278) | -0.50 (0.254) | 0.40 (0.371) | -0.68 (0.091) |
|  |  | *Enterobacterales_unclassified* | 0.21 (0.655) | 0.25 (0.587) | 0.16 (0.737) | 0.18 (0.697) | 0.53 (0.222) | -0.34 (0.451) |
|  |  | *Enterobacteriaceae_unclassified* | -0.09 (0.856) | -0.49 (0.260) | -0.36 (0.433) | 0.06 (0.900) | 0.57 (0.183) | 0.08 (0.872) |
|  |  | *Escherichia-Shigella* | 0.73 (0.062) | 0.70 (0.083) | -0.17 (0.710) | 0.05 (0.909) | 0.65 (0.113) | 0.10 (0.824) |
|  |  | *Pantoea* | -0.26 (0.575) | -0.10 (0.837) | 0.23 (0.620) | 0.18 (0.694) | 0.25 (0.591) | -0.43 (0.331) |
|  |  | *Pasteurellaceae_unclassified* | 0.73 (0.062) | 0.34 (0.455) | -0.28 (0.546) | 0.04 (0.935) | 0.41 (0.364) | 0.56 (0.194) |
|  |  | *Sutterella* | 0.62 (0.141) | 0.77 (0.041) | -0.13 (0.779) | 0.02 (0.960) | 0.20 (0.670) | 0.36 (0.423) |
|  | Spirochaetota | | 0.62 (0.134) | 0.13 (0.782) | -0.09 (0.847) | -0.33 (0.467) | 0.87 (0.011) | -0.26 (0.569) |
|  |  | *Treponema* | 0.63 (0.129) | 0.13 (0.776) | -0.07 (0.874) | -0.32 (0.479) | 0.86 (0.013) | -0.27 (0.552) |
|  | Synergistota | | 0.28 (0.542) | 0.60 (0.151) | 0.14 (0.766) | 0.61 (0.146) | 0.25 (0.584) | 0.04 (0.934) |
|  |  | *Fretibacterium* | 0.10 (0.832) | 0.47 (0.287) | 0.02 (0.964) | 0.72 (0.070) | 0.17 (0.716) | 0.18 (0.693) |
|  | Mycoplasmatota | |  |  |  |  |  |  |
|  |  | *Mycoplasma* | 0.58 (0.168) | 0.29 (0.535) | -0.59 (0.165) | 0.23 (0.612) | 0.60 (0.153) | 0.71 (0.074) |
|  |  |  |  |  |  |  |  |  |
| Inulin | Total bacteria | | 0.58 (0.175) | -0.65 (0.115) | -0.50 (0.251) | -0.29 (0.524) | -0.34 (0.449) | 0.29 (0.534) |
|  | Actinobacteriota | | 0.46 (0.301) | -0.71 (0.071) | -0.72 (0.067) | -0.12 (0.802) | -0.71 (0.073) | 0.35 (0.440) |
|  |  | *Actinomyces* | 0.55 (0.205) | -0.44 (0.319) | -0.49 (0.268) | -0.27 (0.556) | -0.22 (0.630) | 0.47 (0.287) |
|  |  | *Bifidobacterium* | 0.26 (0.575) | -0.50 (0.249) | -0.92 (0.004) | -0.31 (0.495) | -0.58 (0.171) | 0.68 (0.090) |
|  |  | *Collinsella* | 0.58 (0.174) | -0.33 (0.475) | -0.39 (0.392) | -0.69 (0.086) | 0.38 (0.398) | 0.27 (0.553) |
|  | Bacteroidota | | 0.64 (0.118) | -0.61 (0.144) | -0.50 (0.252) | -0.59 (0.161) | 0.02 (0.972) | 0.27 (0.561) |
|  |  | *Alloprevotella* | -0.12 (0.954) | 0.23 (0.814) | 0.68 (0.599) | 0.26 (0.146) | 0.28 (0.147) | -0.56 (0.683) |
|  |  | *Bacteroides* | 0.66 (0.104) | -0.82 (0.024) | -0.49 (0.259) | -0.32 (0.479) | -0.40 (0.374) | 0.09 (0.854) |
|  |  | *Muribaculaceae_unclassified* | 0.48 (0.275) | -0.83 (0.021) | 0.50 (0.249) | -0.81 (0.028) | -0.03 (0.954) | 0.03 (0.957) |
|  |  | *Parabacteroides* | 0.48 (0.272) | -0.50 (0.250) | -0.12 (0.805) | -0.01 (0.979) | -0.29 (0.523) | -0.09 (0.849) |
|  |  | *Porphyromonas* | 0.33 (0.469) | -0.43 (0.330) | -0.20 (0.665) | -0.25 (0.588) | -0.14 (0.757) | 0.16 (0.738) |
|  |  | *Prevotella* | 0.39 (0.388) | -0.09 (0.840) | -0.39 (0.381) | -0.70 (0.077) | 0.46 (0.296) | 0.61 (0.143) |
|  |  | *Prevotellaceae_NK3B31_group* | 0.62 (0.141) | -0.23 (0.625) | -0.44 (0.324) | -0.48 (0.281) | 0.23 (0.614) | 0.46 (0.297) |
|  |  | *Prevotellaceae_UCG003* | -0.23 (0.623) | -0.10 (0.826) | 0.56 (0.191) | -0.19 (0.687) | 0.37 (0.411) | -0.71 (0.074) |
|  |  | *Prevotellaceae_unclassified* | 0.79 (0.033) | -0.73 (0.063) | -0.57 (0.179) | -0.62 (0.140) | -0.07 (0.883) | 0.31 (0.497) |
|  | Firmicutes | | 0.63 (0.130) | -0.59 (0.163) | -0.57 (0.180) | -0.23 (0.628) | -0.40 (0.380) | 0.41 (0.362) |
|  |  | *Agathobacter* | 0.41 (0.364) | 0.14 (0.764) | -0.19 (0.690) | -0.37 (0.413) | 0.51 (0.246) | 0.30 (0.515) |
|  |  | *Anaerovibrio* | 0.07 (0.878) | 0.07 (0.884) | -0.19 (0.682) | -0.35 (0.445) | 0.33 (0.463) | 0.22 (0.640) |
|  |  | *Blautia* | -0.21 (0.644) | 0.33 (0.471) | 0.24 (0.603) | -0.32 (0.488) | 0.66 (0.104) | -0.08 (0.861) |
|  |  | *Clostridium_sensu_stricto_1* | 0.52 (0.229) | -0.53 (0.225) | -0.34 (0.457) | 0.01 (0.990) | -0.42 (0.347) | 0.10 (0.827) |
|  |  | *Enterococcus* | 0.39 (0.387) | -0.17 (0.718) | -0.17 (0.708) | -0.80 (0.029) | 0.58 (0.173) | 0.32 (0.484) |
|  |  | *Faecalibacterium* | -0.18 (0.706) | 0.43 (0.334) | 0.00 (0.994) | -0.31 (0.494) | 0.58 (0.174) | 0.30 (0.510) |
|  |  | *Lachnoanaerobaculum* | 0.47 (0.284) | 0.06 (0.890) | -0.23 (0.625) | -0.40 (0.376) | 0.40 (0.368) | 0.61 (0.147) |
|  |  | *Lachnospiraceae_unclassified* | 0.23 (0.621) | -0.34 (0.458) | 0.03 (0.955) | -0.25 (0.586) | 0.12 (0.804) | -0.20 (0.671) |
|  |  | *Lactobacillus* | -0.37 (0.414) | 0.35 (0.435) | 0.20 (0.673) | 0.70 (0.079) | -0.39 (0.389) | 0.05 (0.914) |
|  |  | *Lactococcus* | -0.14 (0.758) | -0.14 (0.761) | 0.03 (0.957) | 0.12 (0.806) | -0.32 (0.491) | 0.00 (0.994) |
|  |  | *Megamonas* | 0.15 (0.752) | 0.31 (0.495) | -0.39 (0.388) | -0.35 (0.443) | 0.36 (0.422) | 0.75 (0.051) |
|  |  | *Megasphaera* | 0.35 (0.437) | -0.46 (0.296) | -0.87 (0.010) | -0.31 (0.501) | -0.48 (0.277) | 0.71 (0.076) |
|  |  | *Parvimonas* | 0.40 (0.379) | -0.45 (0.310) | -0.13 (0.784) | -0.08 (0.859) | -0.22 (0.640) | -0.01 (0.989) |
|  |  | *Peptostreptococcaceae_unclassified* | 0.50 (0.257) | -0.35 (0.448) | 0.12 (0.801) | 0.10 (0.835) | -0.12 (0.791) | -0.29 (0.523) |
|  |  | *Phascolarctobacterium* | 0.46 (0.298) | -0.37 (0.415) | -0.29 (0.528) | -0.42 (0.345) | 0.14 (0.772) | 0.14 (0.764) |
|  |  | *Romboutsia* | 0.49 (0.260) | -0.59 (0.163) | -0.33 (0.474) | 0.05 (0.915) | -0.54 (0.213) | 0.05 (0.912) |
|  |  | *Sarcina* | 0.68 (0.092) | -0.50 (0.252) | -0.46 (0.295) | -0.33 (0.472) | -0.18 (0.696) | 0.42 (0.351) |
|  |  | *Selenomonadaceae_unclassified* | 0.23 (0.617) | -0.04 (0.934) | -0.46 (0.298) | 0.03 (0.954) | -0.28 (0.548) | 0.67 (0.101) |
|  |  | *Streptococcus* | 0.76 (0.048) | -0.49 (0.270) | -0.45 (0.308) | -0.21 (0.648) | -0.24 (0.610) | 0.37 (0.409) |
|  |  | *Terrisporobacter* | 0.55 (0.204) | -0.49 (0.262) | -0.30 (0.517) | 0.23 (0.625) | -0.58 (0.168) | 0.03 (0.949) |
|  |  | *Turicibacter* | 0.60 (0.156) | -0.83 (0.021) | -0.64 (0.122) | -0.15 (0.756) | -0.69 (0.085) | 0.19 (0.690) |
|  |  | *Veillonella* | 0.41 (0.355) | -0.28 (0.536) | -0.74 (0.056) | -0.38 (0.394) | -0.18 (0.692) | 0.81 (0.028) |
|  | Fusobacteriota | | 0.69 (0.088) | -0.72 (0.066) | -0.37 (0.420) | -0.45 (0.307) | -0.11 (0.818) | 0.02 (0.961) |
|  |  | *Fusobacterium* | 0.69 (0.084) | -0.72 (0.071) | -0.36 (0.434) | -0.45 (0.317) | -0.10 (0.833) | 0.01 (0.981) |
|  |  | *Leptotrichia* | 0.46 (0.295) | -0.53 (0.255) | -0.69 (0.086) | -0.44 (0.325) | -0.30 (0.519) | 0.65 (0.117) |
|  | Proteobacteria | | 0.45 (0.306) | -0.74 (0.056) | -0.16 (0.727) | -0.49 (0.269) | -0.07 (0.881) | -0.23 (0.615) |
|  |  | *Acinetobacter* | -0.12 (0.806) | 0.23 (0.613) | 0.68 (0.096) | 0.26 (0.572) | 0.28 (0.537) | -0.56 (0.192) |
|  |  | *Actinobacillus* | 0.27 (0.551) | -0.26 (0.574) | 0.28 (0.542) | 0.11 (0.808) | 0.00 (0.992) | -0.68 (0.090) |
|  |  | *Enterobacterales_unclassified* | 0.28 (0.536) | -0.51 (0.244) | 0.09 (0.855) | -0.12 (0.804) | -0.15 (0.753) | -0.35 (0.447) |
|  |  | *Enterobacteriaceae_unclassified* | 0.18 (0.692) | 0.10 (0.829) | 0.28 (0.543) | -0.09 (0.855) | 0.32 (0.480) | -0.05 (0.907) |
|  |  | *Escherichia_Shigella* | 0.77 (0.044) | -0.86 (0.014) | -0.57 (0.180) | -0.72 (0.069) | -0.08 (0.864) | 0.17 (0.723) |
|  |  | *Pantoea* | -0.17 (0.715) | -0.12 (0.799) | 0.42 (0.348) | 0.23 (0.625) | -0.12 (0.794) | -0.48 (0.272) |
|  |  | *Pasteurellaceae_unclassified* | 0.77 (0.045) | -0.09 (0.856) | -0.32 (0.487) | -0.45 (0.309) | 0.43 (0.334) | 0.43 (0.330) |
|  |  | *Sutterella* | 0.54 (0.207) | -0.50 (0.248) | -0.60 (0.156) | -0.60 (0.153) | 0.03 (0.955) | 0.38 (0.403) |
|  | Spirochaetota | | 0.55 (0.202) | -0.57 (0.183) | -0.02 (0.964) | -0.47 (0.288) | 0.16 (0.725) | -0.32 (0.484) |
|  |  | *Treponema* | 0.55 (0.198) | -0.57 (0.181) | -0.02 (0.964) | -0.45 (0.305) | 0.15 (0.747) | -0.33 (0.477) |
|  | Synergistota | | 0.48 (0.271) | -0.51 (0.245) | -0.25 (0.591) | -0.13 (0.775) | -0.26 (0.572) | 0.10 (0.834) |
|  |  | *Fretibacterium* | 0.41 (0.367) | -0.35 (0.438) | -0.23 (0.621) | -0.09 (0.850) | -0.22 (0.642) | 0.23 (0.622) |
|  | Mycoplasmatota | |  |  |  |  |  |  |
|  |  | *Mycoplasma* | 0.86 (0.013) | -0.28 (0.547) | -0.50 (0.251) | -0.68 (0.095) | 0.36 (0.425) | 0.64 (0.125) |
|  |  |  |  |  |  |  |  |  |
| Pectin | Total bacteria | | -0.11 (0.814) | 0.46 (0.299) | -0.35 (0.438) | 0.47 (0.282) | -0.28 (0.545) | 0.45 (0.312) |
|  | Actinobacteriota | | -0.16 (0.726) | 0.30 (0.509) | -0.54 (0.207) | 0.24 (0.607) | -0.40 (0.373) | 0.47 (0.288) |
|  |  | *Actinomyces* | -0.35 (0.437) | 0.39 (0.385) | -0.40 (0.376) | 0.27 (0.560) | -0.45 (0.311) | 0.45 (0.311) |
|  |  | *Bifidobacterium* | -0.41 (0.362) | 0.28 (0.539) | -0.72 (0.067) | -0.22 (0.643) | -0.20 (0.671) | 0.64 (0.120) |
|  |  | *Collinsella* | -0.09 (0.855) | -0.08 (0.856) | -0.72 (0.071) | 0.44 (0.326) | 0.13 (0.782) | -0.06 (0.890) |
|  | Bacteroidota | | -0.03 (0.950) | 0.24 (0.598) | -0.62 (0.140) | 0.65 (0.118) | -0.07 (0.877) | 0.22 (0.632) |
|  |  | *Alloprevotella* | 0.41 (0.355) | 0.55 (0.203) | 0.34 (0.462) | 0.59 (0.161) | 0.72 (0.066) | 0.21 (0.645) |
|  |  | *Bacteroides* | -0.03 (0.800) | 0.24 (0.311) | -0.62 (0.497) | 0.65 (0.140) | -0.07 (0.947) | 0.22 (0.416) |
|  |  | *Muribaculaceae_unclassified* | 0.41 (0.364) | 0.58 (0.169) | -0.34 (0.457) | 0.76 (0.048) | 0.67 (0.103) | 0.45 (0.315) |
|  |  | *Parabacteroides* | 0.11 (0.809) | 0.37 (0.413) | 0.09 (0.853) | 0.46 (0.293) | -0.13 (0.780) | 0.20 (0.664) |
|  |  | *Porphyromonas* | 0.00 (0.993) | 0.63 (0.131) | 0.04 (0.938) | 0.47 (0.283) | -0.13 (0.775) | 0.50 (0.257) |
|  |  | *Prevotella* | -0.42 (0.353) | 0.17 (0.717) | -0.64 (0.118) | 0.25 (0.590) | -0.17 (0.712) | 0.26 (0.574) |
|  |  | *Prevotellaceae_NK3B31_group* | -0.38 (0.396) | -0.17 (0.722) | -0.80 (0.032) | 0.26 (0.576) | -0.34 (0.454) | -0.05 (0.921) |
|  |  | *Prevotellaceae_UCG003* | 0.82 (0.022) | 0.35 (0.442) | 0.66 (0.109) | 0.73 (0.061) | 0.73 (0.061) | -0.10 (0.836) |
|  |  | *Prevotellaceae_unclassified* | -0.06 (0.891) | 0.40 (0.371) | -0.55 (0.205) | 0.61 (0.146) | 0.00 (0.993) | 0.36 (0.430) |
|  | Firmicutes | | -0.28 (0.536) | 0.37 (0.412) | -0.44 (0.329) | 0.29 (0.535) | -0.41 (0.364) | 0.44 (0.324) |
|  |  | *Agathobacter* | -0.34 (0.459) | -0.55 (0.203) | -0.67 (0.098) | -0.09 (0.846) | -0.04 (0.937) | -0.41 (0.363) |
|  |  | *Anaerovibrio* | -0.13 (0.776) | -0.36 (0.423) | -0.61 (0.146) | 0.10 (0.825) | -0.08 (0.865) | -0.23 (0.623) |
|  |  | *Blautia* | 0.13 (0.786) | -0.32 (0.486) | -0.17 (0.711) | 0.19 (0.691) | 0.22 (0.636) | -0.36 (0.425) |
|  |  | *Clostridium_sensu_stricto_1* | -0.07 (0.874) | 0.15 (0.743) | -0.30 (0.517) | 0.40 (0.380) | -0.45 (0.310) | 0.15 (0.753) |
|  |  | *Enterococcus* | -0.08 (0.864) | 0.52 (0.228) | -0.09 (0.846) | 0.25 (0.582) | 0.57 (0.186) | 0.39 (0.382) |
|  |  | *Faecalibacterium* | -0.27 (0.551) | -0.36 (0.432) | -0.40 (0.376) | -0.21 (0.652) | 0.00 (0.997) | -0.20 (0.662) |
|  |  | *Lachnoanaerobaculum* | -0.60 (0.151) | 0.12 (0.803) | -0.42 (0.351) | 0.04 (0.926) | -0.44 (0.321) | 0.18 (0.695) |
|  |  | *Lachnospiraceae_unclassified* | 0.31 (0.498) | 0.13 (0.786) | -0.14 (0.766) | 0.79 (0.037) | -0.08 (0.872) | -0.07 (0.889) |
|  |  | *Lactobacillus* | -0.30 (0.514) | -0.13 (0.776) | 0.25 (0.582) | -0.39 (0.385) | -0.80 (0.032) | -0.01 (0.979) |
|  |  | *Lactococcus* | 0.07 (0.882) | 0.65 (0.118) | 0.51 (0.237) | -0.02 (0.964) | 0.08 (0.858) | 0.55 (0.205) |
|  |  | *Megamonas* | -0.73 (0.060) | -0.30 (0.517) | -0.72 (0.068) | -0.47 (0.289) | -0.35 (0.447) | 0.04 (0.939) |
|  |  | *Megasphaera* | -0.48 (0.274) | 0.16 (0.740) | -0.84 (0.017) | -0.09 (0.848) | -0.43 (0.334) | 0.50 (0.248) |
|  |  | *Parvimonas* | 0.07 (0.882) | 0.39 (0.382) | 0.00 (0.993) | 0.53 (0.220) | -0.24 (0.597) | 0.25 (0.593) |
|  |  | *Peptostreptococcaceae_unclassified* | 0.20 (0.670) | 0.15 (0.747) | 0.24 (0.598) | 0.39 (0.392) | 0.02 (0.969) | -0.09 (0.848) |
|  |  | *Phascolarctobacterium* | 0.00 (0.994) | -0.03 (0.943) | -0.57 (0.179) | 0.58 (0.171) | -0.18 (0.693) | -0.05 (0.912) |
|  |  | *Romboutsia* | -0.01 (0.987) | 0.34 (0.454) | -0.10 (0.838) | 0.34 (0.456) | -0.30 (0.512) | 0.30 (0.520) |
|  |  | *Sarcina* | -0.31 (0.505) | 0.49 (0.260) | -0.25 (0.591) | 0.21 (0.651) | -0.17 (0.711) | 0.49 (0.266) |
|  |  | *Selenomonadaceae_unclassified* | -0.68 (0.090) | 0.07 (0.890) | -0.49 (0.260) | -0.23 (0.612) | -0.83 (0.020) | 0.34 (0.460) |
|  |  | *Streptococcus* | -0.34 (0.460) | 0.32 (0.480) | -0.29 (0.532) | 0.13 (0.777) | -0.24 (0.604) | 0.34 (0.457) |
|  |  | *Terrisporobacter* | -0.10 (0.828) | 0.09 (0.849) | -0.13 (0.784) | 0.13 (0.784) | -0.39 (0.393) | 0.10 (0.833) |
|  |  | *Turicibacter* | 0.00 (0.992) | 0.41 (0.357) | -0.36 (0.431) | 0.35 (0.441) | -0.16 (0.730) | 0.46 (0.302) |
|  |  | *Veillonella* | -0.64 (0.124) | 0.16 (0.728) | -0.82 (0.025) | -0.05 (0.991) | -0.57 (0.182) | 0.46 (0.294) |
|  | Fusobacteriota | | 0.17 (0.708) | 0.35 (0.449) | -0.34 (0.455) | 0.75 (0.051) | 0.05 (0.098) | 0.20 (0.668) |
|  |  | *Fusobacterium* | 0.18 (0.703) | 0.32 (0.486) | -0.34 (0.449) | 0.75 (0.052) | 0.05 (0.908) | 0.17 (0.713) |
|  |  | *Leptotrichia* | -0.40 (0.380) | 0.58 (0.174) | -0.50 (0.250) | 0.21 (0.646) | -0.35 (0.442) | 0.71 (0.072) |
|  | Proteobacteria | | 0.51 (0.245) | 0.65 (0.116) | 0.09 (0.851) | 0.86 (0.014) | 0.50 (0.259) | 0.35 (0.446) |
|  |  | *Acinetobacter* | 0.37 (0.412) | 0.08 (0.873) | 0.80 (0.032) | 0.13 (0.778) | 0.34 (0.462) | -0.27 (0.563) |
|  |  | *Actinobacillus* | 0.59 (0.162) | -0.28 (0.538) | 0.22 (0.637) | 0.38 (0.405) | 0.50 (0.255) | -0.54 (0.212) |
|  |  | *Enterobacterales_unclassified* | 0.45 (0.309) | 0.55 (0.203) | 0.37 (0.411) | 0.66 (0.104) | 0.27 (0.565) | 0.23 (0.620) |
|  |  | *Enterobacteriaceae_unclassified* | -0.02 (0.970) | 0.32 (0.489) | 0.46 (0.295) | 0.02 (0.973) | 0.26 (0.577) | 0.10 (0.830) |
|  |  | *Escherichia_Shigella* | 0.16 (0.727) | 0.51 (0.247) | -0.46 (0.302) | 0.72 (0.070) | 0.34 (0.462) | 0.40 (0.376) |
|  |  | *Pantoea* | 0.46 (0.294) | 0.45 (0.306) | 0.73 (0.065) | 0.42 (0.354) | 0.14 (0.759) | 0.13 (0.776) |
|  |  | *Pasteurellaceae_unclassified* | -0.47 (0.289) | -0.33 (0.464) | -0.74 (0.059) | 0.10 (0.826) | -0.24 (0.599) | -0.24 (0.597) |
|  |  | *Sutterella* | -0.13 (0.776) | 0.05 (0.915) | -0.81 (0.027) | 0.44 (0.324) | -0.09 (0.853) | 0.16 (0.739) |
|  | Spirochaetota | | 0.49 (0.269) | 0.37 (0.411) | 0.12 (0.800) | 0.67 (0.097) | 0.69 (0.085) | 0.05 (0.909) |
|  |  | *Treponema* | 0.48 (0.270) | 0.36 (0.424) | 0.12 (0.800) | 0.67 (0.101) | 0.69 (0.089) | 0.05 (0.922) |
|  | Synergistota | | -0.02 (0.972) | 0.40 (0.373) | -0.11 (0.814) | 0.49 (0.267) | -0.28 (0.549) | 0.30 (0.512) |
|  |  | *Fretibacterium* | -0.18 (0.695) | 0.41 (0.364) | -0.08 (0.860) | 0.31 (0.497) | -0.39 (0.385) | 0.36 (0.431) |
|  | Mycoplasmatota | |  |  |  |  |  |  |
|  |  | *Mycoplasma* | -0.53 (0.220) | 0.15 (0.753) | -0.65 (0.115) | 0.13 (0.777) | -0.09 (0.854) | 0.21 (0.648) |
|  |  |  |  |  |  |  |  |  |
| High-amylose starch | Total bacteria | | -0.33 (0.473) | 0.08 (0.866) | -0.58 (0.169) | -0.43 (0.337) | -0.11 (0.809) | 0.17 (0.712) |
|  | Actinobacteriota | | -0.16 (0.732) | -0.03 (0.941) | -0.65 (0.112) | -0.27 (0.563) | -0.52 (0.229) | 0.04 (0.931) |
|  |  | *Actinomyces* | -0.38 (0.394) | 0.09 (0.842) | -0.61 (0.144) | -0.41 (0.362) | -0.08 (0.868) | 0.30 (0.520) |
|  |  | *Bifidobacterium* | 0.32 (0.478) | 0.26 (0.571) | -0.78 (0.041) | -0.40 (0.376) | -0.56 (0.195) | 0.42 (0.351) |
|  |  | *Collinsella* | 0.34 (0.462) | 0.23 (0.621) | -0.59 (0.162) | -0.59 (0.165) | 0.57 (0.180) | 0.12 (0.802) |
|  | Bacteroidota | | -0.07 (0.881) | 0.22 (0.628) | -0.70 (0.080) | -0.58 (0.176) | 0.28 (0.546) | 0.15 (0.755) |
|  |  | *Alloprevotella* | 0.01 (0.990) | 0.62 (0.140) | 0.02 (0.970) | -0.46 (0.296) | 0.70 (0.079) | 0.45 (0.316) |
|  |  | *Bacteroides* | -0.11 (0.810) | 0.00 (0.994) | -0.50 (0.255) | -0.48 (0.274) | -0.07 (0.874) | 0.01 (0.984) |
|  |  | *Muribaculaceae_unclassified* | 0.40 (0.379) | 0.52 (0.235) | -0.55 (0.202) | -0.76 (0.047) | 0.28 (0.543) | 0.30 (0.507) |
|  |  | *Parabacteroides* | -0.35 (0.440) | -0.19 (0.679) | -0.12 (0.799) | -0.21 (0.647) | -0.05 (0.915) | -0.10 (0.831) |
|  |  | *Porphyromonas* | -0.51 (0.240) | 0.23 (0.614) | -0.34 (0.455) | -0.34 (0.451) | 0.01 (0.989) | 0.32 (0.481) |
|  |  | *Prevotella* | -0.08 (0.861) | 0.52 (0.236) | -0.73 (0.062) | -0.58 (0.176) | 0.49 (0.261) | 0.56 (0.191) |
|  |  | *Prevotellaceae_NK3B31_group* | 0.03 (0.952) | 0.06 (0.907) | -0.66 (0.103) | -0.45 (0.317) | 0.37 (0.414) | 0.09 (0.845) |
|  |  | *Prevotellaceae_UCG003* | -0.18 (0.692) | 0.31 (0.502) | 0.42 (0.348) | -0.02 (0.971) | 0.49 (0.261) | -0.07 (0.886) |
|  |  | *Prevotellaceae_unclassified* | 0.03 (0.954) | 0.18 (0.703) | -0.70 (0.082) | -0.71 (0.073) | 0.24 (0.605) | 0.22 (0.637) |
|  | Firmicutes | | -0.26 (0.571) | -0.03 (0.957) | -0.61 (0.147) | -0.42 (0.353) | -0.19 (0.679) | 0.17 (0.715) |
|  |  | *Agathobacter* | 0.53 (0.225) | -0.08 (0.869) | -0.30 (0.509) | -0.30 (0.518) | 0.54 (0.210) | -0.06 (0.894) |
|  |  | *Anaerovibrio* | 0.18 (0.700) | 0.20 (0.668) | -0.38 (0.398) | -0.13 (0.785) | 0.32 (0.486) | 0.01 (0.979) |
|  |  | *Blautia* | 0.10 (0.837) | 0.33 (0.476) | -0.02 (0.970) | 0.00 (0.998) | 0.59 (0.167) | 0.03 (0.946) |
|  |  | *Clostridium_sensu_stricto_1* | -0.40 (0.380) | -0.24 (0.599) | -0.37 (0.408) | -0.15 (0.748) | -0.20 (0.670) | -0.18 (0.701) |
|  |  | *Enterococcus* | 0.36 (0.423) | 0.58 (0.168) | -0.33 (0.466) | -0.79 (0.033) | 0.67 (0.099) | 0.70 (0.083) |
|  |  | *Faecalibacterium* | 0.23 (0.618) | 0.33 (0.469) | -0.20 (0.661) | -0.06 (0.891) | 0.40 (0.368) | 0.24 (0.602) |
|  |  | *Lachnoanaerobaculum* | -0.29 (0.534) | 0.19 (0.687) | -0.51 (0.239) | -0.43 (0.333) | 0.41 (0.357) | 0.45 (0.310) |
|  |  | *Lachnospiraceae_unclassified* | -0.49 (0.263) | 0.14 (0.772) | -0.24 (0.598) | -0.12 (0.793) | 0.31 (0.499) | -0.14 (0.770) |
|  |  | *Lactobacillus* | -0.76 (0.047) | -0.33 (0.474) | 0.21 (0.648) | 0.63 (0.133) | -0.58 (0.169) | -0.16 (0.725) |
|  |  | *Lactococcus* | -0.31 (0.495) | 0.17 (0.714) | 0.12 (0.797) | -0.03 (0.951) | -0.34 (0.459) | 0.34 (0.457) |
|  |  | *Megamonas* | 0.28 (0.537) | 0.23 (0.6262) | -0.53 (0.221) | -0.27 (0.563) | 0.20 (0.666) | 0.42 (0.347) |
|  |  | *Megasphaera* | 0.10 (0.837) | 0.19 (0.675) | -0.85 (0.015) | -0.36 (0.427) | -0.43 (0.331) | 0.32 (0.478) |
|  |  | *Parvimonas* | -0.54 (0.210) | -0.03 (0.946) | -0.24 (0.601) | -0.20 (0.674) | -0.01 (0.975) | 0.00 (0.995) |
|  |  | *Peptostreptococcaceae_unclassified* | -0.16 (0.735) | -0.41 (0.362) | 0.17 (0.722) | -0.13 (0.780) | 0.13 (0.785) | -0.31 (0.494) |
|  |  | *Phascolarctobacterium* | -0.17 (0.714) | 0.12 (0.802) | -0.53 (0.216) | -0.32 (0.477) | 0.33 (0.472) | -0.05 (0.924) |
|  |  | *Romboutsia* | -0.33 (0.473) | -0.24 (0.608) | -0.27 (0.562) | -0.18 (0.698) | -0.31 (0.501) | -0.11 (0.817) |
|  |  | *Sarcina* | -0.10 (0.839) | 0.04 (0.939) | -0.48 (0.270) | -0.57 (0.182) | 0.01 (0.978) | 0.34 (0.461) |
|  |  | *Selenomonadaceae_unclassified* | -0.46 (0.301) | -0.01 (0.990) | -0.56 (0.190) | -0.07 (0.882) | -0.34 (0.453) | 0.28 (0.546) |
|  |  | *Streptococcus* | -0.01 (0.985) | -0.18 (0.707) | -0.42 (0.354) | -0.50 (0.249) | -0.02 (0.965) | 0.16 (0.737) |
|  |  | *Terrisporobacter* | -0.16 (0.724) | -0.51 (0.241) | -0.14 (0.758) | -0.07 (0.876) | -0.37 (0.419) | -0.31 (0.501) |
|  |  | *Turicibacter* | -0.03 (0.951) | -0.12 (0.799) | -0.51 (0.245) | -0.38 (0.407) | -0.41 (0.356) | -0.02 (0.970) |
|  |  | *Veillonella* | -0.13 (0.785) | 0.26 (0.580) | -0.87 (0.010) | -0.42 (0.354) | -0.17 (0.717) | 0.45 (0.308) |
|  | Fusobacteria | | -0.10 (0.828) | 0.06 (0.900) | -0.48 (0.276) | -0.53 (0.224) | 0.23 (0.622) | -0.01 (0.980) |
|  |  | *Fusobacterium* | -0.09 (0.848) | 0.04 (0.935) | -0.47 (0.290) | -0.52 (0.232) | 0.24 (0.604) | -0.04 (0.940) |
|  |  | *Leptotrichia* | -0.28 (0.550) | 0.36 (0.428) | -0.80 (0.031) | -0.54 (0.214) | -0.19 (0.684) | 0.56 (0.194) |
|  | Proteobacteria | | -0.05 (0.916) | 0.26 (0.568) | -0.23 (0.626) | -0.53 (0.218) | 0.26 (0.576) | 0.11 (0.810) |
|  |  | *Acinetobacter* | -0.16 (0.734) | -0.23 (0.628) | 0.69 (0.084) | 0.19 (0.690) | 0.31 (0.496) | -0.22 (0.643) |
|  |  | *Actinobacillus* | 0.43 (0.342) | -0.50 (0.257) | 0.44 (0.321) | 0.03 (0.942) | 0.26 (0.575) | -0.69 (0.084) |
|  |  | *Enterobacterales_unclassified* | -0.32 (0.491) | 0.03 (0.954) | 0.06 (0.899) | -0.22 (0.628) | 0.10 (0.828) | -0.04 (0.937) |
|  |  | *Enterobacteriaceae_unclassified* | -0.01 (0.977) | 0.00 (0.993) | 0.26 (0.568) | -0.23 (0.614) | 0.36 (0.429) | 0.23 (0.620) |
|  |  | *Escherichia_Shigella* | 0.25 (0.423) | 0.27 (0.168) | -0.63 (0.128) | -0.80 (0.032) | 0.28 (0.099) | 0.22 (0.083) |
|  |  | *Pantoea* | -0.58 (0.168) | 0.00 (1.00) | 0.39 (0.383) | 0.18 (0.704) | -0.05 (0.917) | -0.08 (0.871) |
|  |  | *Pasteurellaceae_unclassified* | 0.28 (0.541) | -0.14 (0.758) | -0.49 (0.260) | -0.50 (0.251) | 0.58 (0.173) | 0.01 (0.985) |
|  |  | *Sutterella* | 0.14 (0.757) | 0.25 (0.589) | -0.76 (0.046) | -0.53 (0.222) | 0.22 (0.639) | 0.15 (0.751) |
|  | Spirochaetota | | 0.35 (0.442) | 0.06 (0.900) | -0.01 (0.983) | -0.56 (0.193) | 0.49 (0.259) | -0.04 (0.938) |
|  |  | *Treponema* | 0.35 (0.437) | 0.04 (0.932) | 0.00 (0.994) | -0.55 (0.201) | 0.48 (0.272) | -0.05 (0.912) |
|  | Synergistota | | -0.46 (0.295) | -0.03 (0.941) | -0.34 (0.452) | -0.27 (0.552) | -0.05 (0.922) | 0.05 (0.923) |
|  |  | *Fretibacterium* | -0.53 (0.217) | 0.00 (0.997) | -0.34 (0.456) | -0.24 (0.611) | -0.08 (0.864) | 0.17 (0.714) |
|  | Mycoplasmatota | |  |  |  |  |  |  |
|  |  | *Mycoplasma* | 0.29 (0.528) | 0.13 (0.776) | -0.66 (0.108) | -0.80 (0.029) | 0.53 (0.219) | 0.42 (0.345) |
| ^1^ Values are correlation coefficients with *P* values between parentheses. Only taxa with >1% relative abundance in at least one sample were considered. Bacteria data were log_10_ transformed to achieve homogenous variance. AG, arabinogalactan; DM, dry matter; FOS, fructooligosaccharides; OM, organic matter. | | | | | | | | |

## Supplementary Figure





**Supplementary Figure 1:** Shannon diversity of the microbial community in ileal digesta from growing pigs fed diets for seven days containing different test foods. Data points represent individual samples. The line represents the mean, *n* = 4-6 per diet. The effect of the diet was assessed using a one-way ANOVA test (*P* = 0.665).
